# Full-length autonomous transposable elements are preferentially targeted by expression-dependent forms of RNA-directed DNA methylation

**DOI:** 10.1186/s13059-016-1032-y

**Published:** 2016-08-09

**Authors:** Kaushik Panda, Lexiang Ji, Drexel A. Neumann, Josquin Daron, Robert J. Schmitz, R. Keith Slotkin

**Affiliations:** 1 Department of Molecular Genetics, The Ohio State University, Columbus, OH USA; 2 Institute of Bioinformatics, University of Georgia, Athens, GA USA; 3 Department of Genetics, University of Georgia, Athens, GA USA

**Keywords:** Cytosine methylation, MethylC-seq, RNA-directed DNA methylation (RdDM), Transposable element (TE), Small interfering RNA (siRNA), RNA interference (RNAi), Methylome, TE-silent context, TE-active context, Decrease in DNA methylation 1 (DDM1)

## Abstract

**Background:**

Chromatin modifications such as DNA methylation are targeted to transposable elements by small RNAs in a process termed RNA-directed DNA methylation (RdDM). In plants, canonical RdDM functions through RNA polymerase IV to reinforce pre-existing transposable element silencing. Recent investigations have identified a “non-canonical” form of RdDM dependent on RNA polymerase II expression to initiate and re-establish silencing of active transposable elements. This expression-dependent RdDM mechanism functions through RNAi degradation of transposable element mRNAs into small RNAs guided by the RNA-dependent RNA polymerase 6 (RDR6) protein and is therefore referred to as RDR6-RdDM.

**Results:**

We performed whole-genome MethylC-seq in 20 mutants that distinguish RdDM mechanisms when transposable elements are either transcriptionally silent or active. We identified a new mechanism of expression-dependent RdDM, which functions through DICER-LIKE3 (DCL3) but bypasses the requirement of both RNA polymerase IV and RDR6 (termed DCL3-RdDM). We found that RNA polymerase II expression-dependent forms of RdDM function on over 20 % of transcribed transposable elements, including the majority of full-length elements with all of the domains required for autonomous transposition. Lastly, we find that RDR6-RdDM preferentially targets long transposable elements due to the specificity of primary small RNAs to cleave full-length mRNAs.

**Conclusions:**

Expression-dependent forms of RdDM function to critically target DNA methylation to full-length and transcriptionally active transposable elements, suggesting that these pathways are key to suppressing mobilization. This targeting specificity is initiated on the mRNA cleavage-level, yet manifested as chromatin-level silencing that in plants is epigenetically inherited from generation to generation.

**Electronic supplementary material:**

The online version of this article (doi:10.1186/s13059-016-1032-y) contains supplementary material, which is available to authorized users.

## Background

Transposable elements (TEs) are mobile fragments of DNA that can generate mutations and genome instability. To repress TE activity and new mutations, cells target TEs for epigenetic transcriptional silencing. Small RNAs (sRNAs) are the triggers of epigenetic transcriptional silencing targeted to transposable elements (TEs) and transgenes. sRNAs are known to direct cytosine DNA methylation and histone tail post-translational modifications in both mice and plants, while in organisms that lack cytosine DNA methylation (such as fission yeast, *C. elegans*, and Drosophila) sRNAs direct only histone tail modifications (reviewed in [1]). The mechanism of small RNA-directed DNA methylation (RdDM) has been extensively investigated in the reference plant Arabidopsis, where a “canonical” form of RdDM has been uncovered (reviewed in [2]). This canonical form of RdDM begins with the transcription of the target locus by the RNA polymerase protein Pol IV, a plant-specific Pol II paralog [3], which generates a non-coding RNA that is immediately converted into double-stranded RNA (dsRNA) via RNA-dependent RNA polymerase 2 (RDR2). The Pol IV/RDR2 derived dsRNA is cleaved by the RNaseIII DICER protein DCL3 into 23–24 nucleotide (nt) small interfering RNAs (siRNAs) and these 24 nt siRNAs are incorporated into either the Argonaute 4 (AGO4) or AGO6 proteins [4]. In the nucleus, the siRNA-loaded AGO4/AGO6 can base pair with a nascent non-coding RNA still attached to its DNA template produced by Pol V, a second plant-specific paralog of Pol II. The Pol V transcript acts as a scaffold for protein assembly, and interaction between AGO4/6 and the Pol V transcript results in the recruitment of the protein DRM2 to methylate the cytosines of the corresponding locus.

Pol IV is recruited to and transcribes regions of the genome that have reduced histone acetylation, undergo CG-context maintenance methylation, and are enriched for H3K9me2 [5, 6], heterochromatic marks that decorate regions of the genome inhibited for mRNA production. Canonical Pol IV-targeted RdDM (Pol IV-RdDM) is known to reinforce DNA methylation at regions of TE heterochromatin adjacent to genes [7, 8]. Several laboratories have recently investigated how DNA methylation is initiated at a region of the genome that is actively producing an mRNA and is not already silenced. These investigations have uncovered various “non-canonical” mechanisms of RdDM, which do not rely on Pol IV, but rather are triggered by Pol II mRNA transcripts [9–13]. Pol II TE mRNAs can undergo degradation via endogenous RNAi into 21–22 nt siRNAs [14, 15]. In Arabidopsis, the TE mRNA is converted into dsRNA via RDR6, and this dsRNA is cleaved into 21–22 nt siRNAs via DCL4 and DCL2, respectively [15]. Originally thought to be only a post-transcriptional mechanism of silencing, several studies have determined that the degradation products of Pol II-derived mRNAs can trigger RdDM [9, 12, 13, 16]. The best characterized of these pathways is RDR6-RdDM, where the RDR6-dependent 21–22 nt siRNAs are incorporated into the AGO6 protein and drive RdDM in a Pol V and DRM2-dependent manner [16].

There are only a few known targets of RDR6-RdDM [12, 16]. This is due to the fact that this pathway acts on Pol II transcriptionally active regions of the genome and over time these regions become transcriptionally silenced and regulated by either Pol IV-RdDM or by the maintenance methylation pathway that is not dependent on small RNAs [7, 17]. Maintenance methylation occurs separately for each cytosine sequence context, with CG methylation propagated by MET1, CHG (where H = A, C or T) by CMT3, and CHH context methylation by CMT2 [17–19]. Like Pol IV, CMT2 and CMT3 are guided to previously silenced loci by the H3K9me2 heterochromatic mark [17, 20]. CHH context maintenance methylation is low compared to CG or CHG [17] and therefore RdDM (which targets all cytosine contexts equally) has traditionally been assayed by investigating the CHH methylation level [21, 22].

Maintenance methylation of TEs is coordinated by Decrease in DNA methylation 1 (DDM1) [23], a *swi/snf* family chromatin remodeling protein. DDM1 specifically regulates TEs and in *ddm1* mutants TEs undergo loss of H3K9me2, CG DNA methylation, and heterochromatin condensation [23, 24]. This results in genome-wide TE transcriptional activation [23] and the triggering of the RNAi mechanism to degrade TE mRNAs into 21–22 nt siRNAs [15, 25]. In *ddm1* mutant plants, TE transcriptional silencing cannot be regained and therefore the cell is stuck in a perpetual cycle of attempted re-silencing via RdDM. Re-targeting of TEs for silencing, and in particular CHH hyper-methylation, is a conserved consequence of TE activation via *ddm1* mutation in Arabidopsis, maize, and rice [12, 26, 27]. *ddm1* mutants display unmatched resolution of the mechanisms the cell uses to re-silence TEs [28, 29]. Investigation of *ddm1* mutants lead to the discovery of RDR6-RdDM [12, 16]; however, the genome-wide roles RDR6-RdDM have been a continued question. For example, what are the additional targets and the overall role of RDR6-RdDM, is this the sole non-canonical RdDM mechanism that functions genome-wide, and why are particular TEs targeted to undergo non-canonical forms of RdDM while others are not? To address these questions, we created a genome-wide DNA methylation and small RNA dataset in 20 key RdDM mutants that span both the TE-silent and TE-active contexts, providing insight to the pathways the plant uses to target DNA methylation to specific TEs.

## Results

### RDR6-RdDM targets many transcriptionally active TEs

The switch from an epigenetically silenced state to transcriptional activation is known to trigger Pol II expression-dependent mechanisms of TE silencing such as RDR6-RdDM on the single-locus level [12]. To examine genome-wide methylation states of both active and inactive TEs, we generated a dataset containing whole-genome MethylC-seq of nine key RdDM mutant genotypes in the wild-type Columbia (wt Col) background as well as the same nine mutant genotypes in the *ddm1* mutant background. TE transcription is globally reactivated in the *ddm1* mutant (Additional file 1: Figure S1) [23], whereas the  RdDM mutants that we investigated generally do not show TE transcriptional reactivation or at least not nearly as severe of a transcriptional reactivation compared to *ddm1*. For example, even in *pol V* mutants, which are defective for all RdDM [30], global TE activation is minimal compared to *ddm1* (Additional file 1: Figure S1) [19, 22]. Therefore, in this study any genotype without *ddm1* is referred to as the TE-silent context and our dataset distinguishes RdDM types in both the TE-silent context and the globally reactivated *ddm1* TE-active context.

**Fig. 1:**
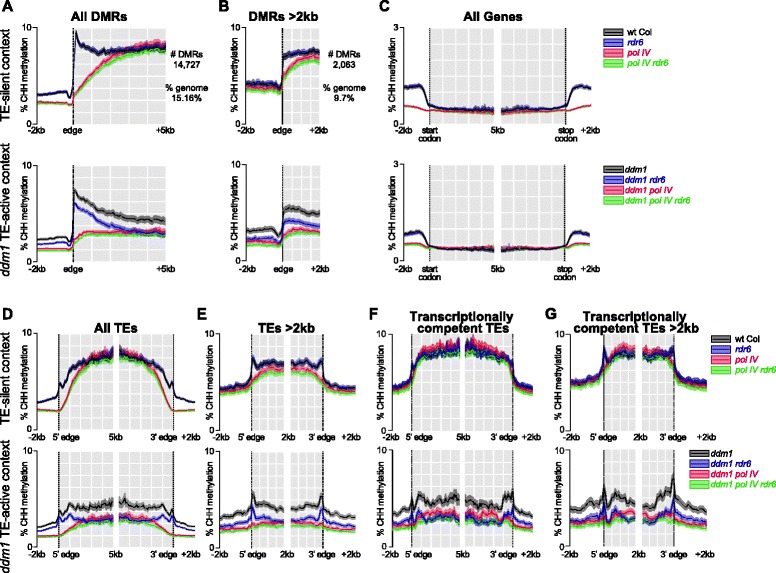
*Meta-plots* of CHH methylation levels in TE-silent and TE-active contexts. **a** Average CHH methylation percentage across all DMRs identified in the TE-silent (*top*) or *ddm1* TE-active (*bottom*) contexts. **b** Analysis of DMRs longer than 2 kb. **c** Alignment of all genes by their 5′ start and 3′ stop codons. **d** Alignment of all TEs by their 5′ and 3′ annotated boundaries. Orientation of the TE was determined using the TAIR10 TE annotation. **e** Alignment of all TEs longer than 2 kb. **f** Alignment of the transcriptionally competent subset of 2374 TEs. **g** Alignment of the transcriptionally competent TEs longer than 2 kb. *Solid lines* represent the 100 bp binned average CHH methylation percentages. The variation of individual element data points is represented as the *transparent colored region around the solid lines* (95 % confidence interval of the average)

We determined that using only uniquely mapping sequencing reads resulted in reduced coverage of repetitive TE regions; however, sequencing coverage was high enough to assay RdDM dynamics of individual TE copies (see “Methods,” Additional file 2: Results, and Additional file 3: Figure S2). To identify the regions of the genome targeted by RDR6-RdDM (and contrast them to the regions regulated by Pol IV-RdDM), we identified differentially methylated regions (DMRs) between all of the genotypes (see “Methods”). Aligning the DMRs, we find that the average wt Col and *rdr6* CHH methylation patterns are indistinguishable, demonstrating that RDR6-RdDM plays a minor genome-wide role in the TE-silent context (Fig. 1a, replicate data in Additional file 4: Figure S3A). In contrast, *pol IV* mutants lose methylation from the DMRs, confirming that Pol IV-RdDM functions to target CHH methylation on a genome-wide level in the TE-silent context (Fig. 1a) [22, 31]. In addition, we assayed the loss of methylation when both RDR6- and Pol IV-RdDM are lost (in *pol IV rdr6* double mutants) and found that this methylation level is slightly reduced compared to the *pol IV* single mutant (Fig. 1a), demonstrating that RDR6-RdDM plays a minor role when Pol IV-RdDM is mutated (see section below on RdDM compensation). In the *ddm1* TE-active context, the overall CHH methylation level is reduced compared to the TE-silent context (Fig. 1a, replicate data in Additional file 4: Figure S3A) [19]. In addition, the *ddm1 rdr6* double mutant shows lower CHH methylation compared to the *ddm1* single mutant (Fig. 1a, replicate data in Additional file 4: Figure S3A), demonstrating a genome-wide role for RDR6-RdDM when TEs are reactivated.

In both the TE-silent and *ddm1* TE-active contexts, loss of CHH methylation in *pol IV* mutants is reduced near the edge of the DMR and less so in the center of the DMR (Fig. 1a). To determine if this loss is due to Pol IV-RdDM functioning specifically at edges of long DMRs or if this effect is due to Pol IV-RdDM’s preference for short TE targets [19], we investigated only DMRs over 2 kb. We found that in the TE-silent context Pol IV-RdDM functions preferentially on long DMR edges, as the CHH methylation in *pol IV* mutants is lost more at the edge compared to the center of a >2 kb DMR (Fig. 1b). At the same time, we found the peak of high CHH methylation at the DMR edge (compared to the body of the DMR) in wt Col and *ddm1* is a function of small DMRs in our analysis, as when only DMRs >2 kb are assayed, the CHH methylation values in wt Col or *ddm1* are consistent over the length of the entire DMR (compare Fig. 1a to 1b, replicate data in Additional file 4: Figure S3A, B). Therefore, at least in the TE-silent context, Pol IV-RdDM targets short DMRs as well as the edges of long DMRs.

A DMR is a computationally identified feature that may span multiple TEs and genes or which may be as short as 4 bp. We found that analysis of the alignment of CHH methylation states of annotated genomic features (such as genes or TEs) was more informative than an analysis of the arbitrary edges of DMRs. For genes, we find that there is low average CHH methylation that is unaltered by Pol IV- or RDR6-RdDM, and we confirm that Pol IV-RdDM is responsible for gene-flanking methylation [22, 32], while RDR6-RdDM does not act near genes (Fig. 1c). For TEs, similar to our findings with DMRs, we find that *rdr6* shows a CHH methylation loss only in the *ddm1* TE-active context but not the TE-silent context (Fig. 1d, replicate data in Additional file 4: Figure S3D). We also observed that loss of CHH methylation in *ddm1 rdr6* mutants occurs not specifically at the edge (as with Pol IV-RdDM at TE edges, see Fig. 1d), but rather acts over the length of the entire long TE and mostly from the TE internal region (Fig. 1e, replicate data in Additional file 4: Figure S3E). Interestingly, in the TE-active context Pol IV-RdDM acts like RDR6-RdDM throughout the length of the entire >2 kb TE (Fig. 1e). We observed this differential role of Pol IV-RdDM with DMRs as well (Fig. 1b) and these data demonstrate that the function of Pol IV-RdDM to reinforce silencing at short TEs and TE edges expands to silencing TE internal body coding regions when TEs are activated. In addition, for TEs >2 kb we find that the *pol IV rdr6* double mutant has lower CHH methylation levels compared to either the *rdr6* or *pol IV* mutants in either the TE-silent or TE-active context (Fig. 1d, e). This demonstrates that the finding on the single-locus level that some TEs are subject to both Pol IV- and RDR6-RdDM to direct full TE CHH methylation [12, 16] is also true on the genome-wide level.

To assess the role of Pol II expression on RdDM dynamics, we focused our analysis on transcriptionally competent TEs by identifying elements with direct evidence of mRNA production in *ddm1* mutant plants (see “Methods”). For this set of 2374 TEs (7.6 % of all TEs) in the TE-silent context, we find that RDR6-RdDM does not function and Pol IV-RdDM’s role is reduced and primarily contributes to the edges of long TEs (Fig. 1f, g, replicate data in Additional file 4: Figure S3F, G). When this set of TEs is specifically transcribed, we find that RDR6-RdDM plays a larger role in TE methylation compared to Pol IV-RdDM, and this is pronounced in the internal regions of long TEs. Therefore, we conclude that RDR6-RdDM targets transcriptionally active TEs on the genome-wide level.

### Dataset capture of both Dicer-dependent and Dicer-independent RdDM

**Fig. 2:**
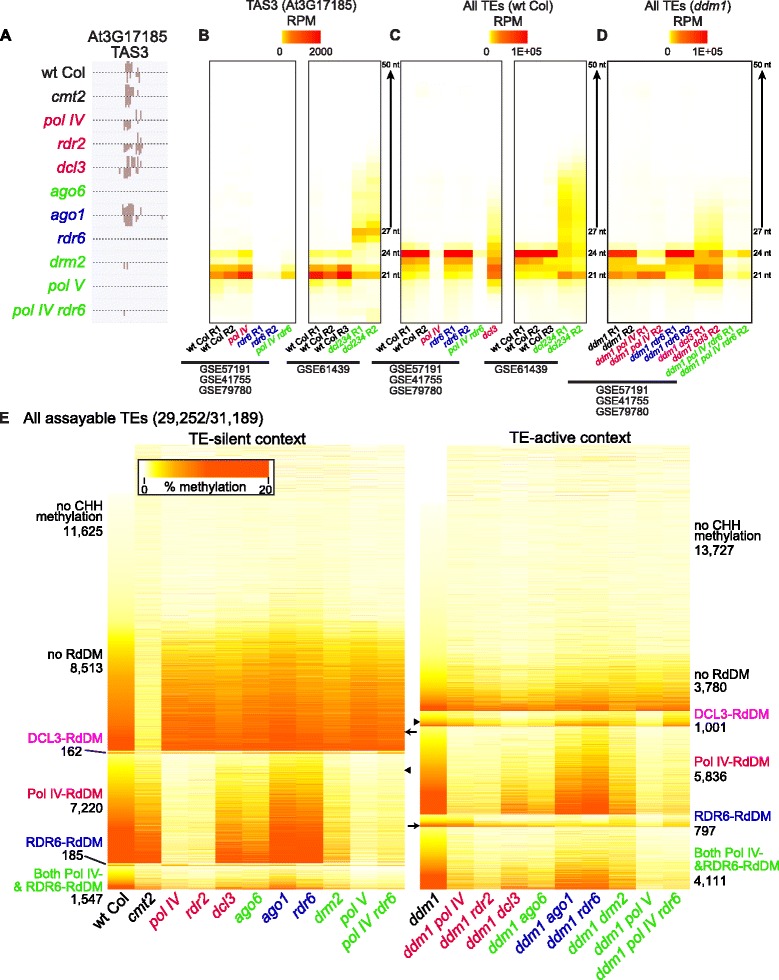
Genome-wide distribution of TE CHH methylation pathways. **a** CHH methylation levels of the RDR6-RdDM targeted locus TAS3. Track scale is 0–50 %. **b** TAS3 small RNA size and abundance *heat maps* in the TE-silent context. *R1*, *R2*, and *R3* signify independent biological replicates. **c** Small RNA size and abundance *heat maps* for all perfectly and uniquely mapping TE small RNAs in the TE-silent context. **d** Small RNA size and abundance *heat maps* for all perfectly and uniquely mapping TE small RNAs in the TE-active context. *GSE numbers* represent the data source. **e** *Heat map* representing CHH methylation values for individual TEs (*rows*). The *columns* represent different genotypes assayed. *Numbers* represent the amount of TEs targeted by each corresponding CHH methylation pathway. TEs change position between the TE-silent (*left*) and *ddm1* TE-active (*right*) panels. The location of the *Athila6A* TE analyzed in Additional file 3: Figure S2C is shown as an *arrow*, while the TE shown in Fig. 3a is marked with an *arrowhead*. Genotypes are color-coded based on the methylation pathway (*black* = maintenance methylation (no RdDM), *red* = Pol IV-RdDM, *blue* = RDR6-RdDM, *green* = contributes to both Pol IV-RdDM and RDR6-RdDM)

Recent data have demonstrated that RdDM can occur through a Dicer-independent mechanism by which either transcribed or processed un-Diced RNAs of ~30–40 nt are trimmed into various small RNA sizes including 21–24 nt siRNAs [33–36]. This Dicer-independent production of small RNAs was shown to occur on both Pol IV and Pol II derived transcripts. While Dicer-dependent production generates specific siRNA size classes, Dicer-independent siRNA production creates small RNAs of all sizes, known as small RNA laddering [35]. We aimed to remove all Dicer-dependent and Dicer-independent TE RdDM at the same time by using a *pol IV rdr6* double mutant. The *pol IV* mutation abolishes Pol IV transcript accumulation upstream of Dicer-independent or Dicer-dependent siRNA production [33, 36]. Because we cannot mutate Pol II’s function without affecting essential non-RdDM networks, we mutated *rdr6* to block the production of dsRNA from Pol II transcripts. By using siRNA laddering as a consequence of Dicer-independent siRNA production, we find that loci that undergo Pol II-dependent RdDM require RDR6 production of dsRNA before either Dicer-dependent or Dicer-independent RdDM (Fig. 2). For example, the TAS3 locus loses CHH methylation in *rdr6* but not *pol IV* mutants (Fig. 2a), confirming that the TAS3 locus is a target of RDR6-RdDM in the TE-silent context [9]. When RDR6 is functional and DCL2, DCL3, and DCL4 are mutated, Dicer-independent processing occurs and generates a ladder of TAS3 siRNA sizes (Fig. 2b) [34, 35]. However, when RDR6 is non-functional (in the *rdr6* mutant), TAS3 siRNAs and laddering are not produced, demonstrating that RDR6 is upstream of Dicer-independent processing (Fig. 2b). The same is true of TE siRNAs: in the TE-silent context they are all dependent on Pol IV (Fig. 2c) and in the TE-active context siRNA laddering does not occur in the *ddm1 pol IV rdr6* triple mutant as it does in *ddm1 dcl3* (Fig. 2d). This result demonstrates that like Pol IV activity, RDR6 activity on TE mRNAs occurs before the Dicer-independent siRNA production that generates siRNA laddering. Therefore, the *pol IV rdr6* double mutant represents the removal of the majority of the upstream dsRNA that drives Dicer-dependent or Dicer-independent RdDM in either the TE-silent or TE-active context. Correspondingly, we find that Pol IV and RDR6 are responsible for nearly all TE RdDM in the TE-silent or TE-active contexts (Fig. 1d, e, Additional file 2: Results, and Additional file 5: Figure S4A), but this level is not 100 % as we have identified a distinct pathway of TE RdDM that is not dependent on either Pol IV or RDR6 (see below).

### Upon TE transcriptional activation, three RdDM mechanisms target genome-wide TE methylation

To characterize the methylation pathways that act on each TE genome-wide, we calculated the CHH methylation level for each of the annotated TE elements and fragments in the Arabidopsis genome (31,189). We were able to successfully cover and individually assay 29,252 (93.8 %) of all TEs, with the majority of TEs lost representing small high-copy TE fragments. We grouped TEs by their mechanism of CHH methylation: no CHH methylation, Pol IV-RdDM (dependent on Pol IV), RDR6-RdDM (dependent on RDR6), and maintenance methylation (not dependent on any RdDM) (see “Methods”) (Fig. 2e). The corresponding CG and CHG methylation analysis is shown in Additional file 6: Figure S5 and replicate data of CHH methylation patterns for key genotypes is shown in Additional file 4: Figure S3H. Similar to the TEs that have been individually investigated and determined to be targets of RDR6-RdDM [12, 16], we found that both RDR6-RdDM and Pol IV-RdDM can target the same TE, providing a distinct co-regulated category (Fig. 2e). In addition, we identified a category of TEs that are methylated by a new pathway of DCL3-dependent 24 nt siRNAs which are not produced from Pol IV, a pathway we refer to as DCL3-RdDM (see below).

Genome-wide distribution of TE CHH methylation in the TE-silent context demonstrates that roughly one-third of TEs do not have CHH methylation, roughly one-third of TEs are not going through RdDM and are subject to only maintenance CHH methylation via CMT2, and roughly one-third are regulated by Pol IV-RdDM (Fig. 2e). This confirms that when TEs are silenced, maintenance methylation and Pol IV-RdDM are the major pathways that mutually exclusively target TE CHH methylation [7]. We find that *pol IV* and *rdr2* mutants have less TE CHH methylation than *dcl3* mutants (Fig. 2e), supporting the Dicer-independent function of Pol IV/RDR2-derived siRNAs in RdDM [35, 36]. On a genome-wide level very few TEs are targeted by DCL3-RdDM or RDR6-RdDM in the TE-silent context, although this number is not zero and we have previously identified a TE that is subject to RDR6-RdDM in wt Col [16]. Consequently, very few TEs in the TE-silent context are regulated by AGO1 and the TEs regulated by AGO6 are targeted through 24 nt siRNAs and the Pol IV-RdDM pathway (Fig. 2e). In addition, we find evidence of 1547 TEs that are primarily targeted by Pol IV-RdDM, but upon loss of Pol IV, these TEs have low levels of RDR6-dependent CHH methylation, demonstrating that they are acted upon by both Pol IV-RdDM and RDR6-RdDM (Fig. 2e). By analyzing mutants that at the same time lose both Pol IV- and RDR6-RdDM, we are able to detect that these two distinct pathways do not function completely independently, but rather one can compensate for the loss of the other (Additional file 7: Figure S6).

We find on the genome-wide level that RdDM regulates more TEs when they lose transcriptional silencing and this is due to an increased number of TEs targeted by the Pol II expression-dependent RdDM pathways (Fig. 2e) (Additional file 5: Figure S4B). Compared to the TE-silent context, in the TE-active context we observe in our dataset an increase in the number of TEs that go through RDR6-RdDM (4.3-fold higher in Fig. 2e), DCL3-RdDM (6.2-fold higher), and the Pol IV-/RDR6- co-regulated RdDM category (2.7-fold higher). We find that roughly one-third (31.6 %) of TEs with CHH methylation in the TE-active context are regulated by RDR6-RdDM (either RDR6-RdDM alone or co-regulated with Pol IV-RdDM). We used the TEs identified in our analysis as regulated by RDR6-RdDM to investigate a replicate dataset and determined that ~50 % of TEs are not covered in the replicate dataset, ~25 % display RDR6-dependent methylation in both datasets, and ~25 % failed to replicate (Additional file 4: Figure S3I). The fraction of RDR6-RdDM TEs that could not be replicated may either be false positives in our analysis or bona fide RDR6-RdDM targets identified but not replicated as a result of the four-fold increase in TE methylation resolution between datasets (Additional file 4: Figure S3J) due to our improved TE mappability (see “Methods” and Additional file 2: Results). Our data prove that RDR6-RdDM does not just function on three TEs (as previously shown [12, 16]), but rather hundreds of individual TEs, and this pathway was likely previously not identified due to the lack of transcriptionally active TEs in wt Col and the activity of Pol IV-RdDM on many of the same TE target loci. A corresponding reduction (we find 2.3-fold) takes place in the number of TEs regulated by maintenance methylation in the *ddm1* TE-active context, demonstrating that DDM1 functions in preserving maintenance methylation-based transcriptional silencing [19]. Of the TEs that undergo any type of RdDM in the TE-silent context, the majority (we find 71.9 %) still undergo RdDM in the *ddm1* TE-active context, while the other TEs lose CHH methylation completely (Additional file 5: Figure S4C). Pol IV-RdDM is still the major RdDM mechanism targeting TEs in *ddm1*, as the number of TEs that undergo Pol IV-RdDM (without RDR6-RdDM) in the *ddm1* background is roughly equal (we find a 1.3-fold change) compared to TE-silent context (and we find a 1.1-fold change when the RDR6 co-regulated pathway is considered). When focused on only transcriptionally competent TEs in the TE-active context, the Pol II-expression dependent RdDM pathways play a pronounced role: we find RDR6-RdDM is 17.4-fold higher, DCL3-RdDM is 18.4-fold higher, and the co-regulated RDR6- and Pol IV-RdDM pathway is 3.6-fold higher compared to the TE-silent context. At the same time, Pol IV-RdDM has decreased function on transcriptionally active TEs (we find a 0.6-fold change) (Additional file 8: Figure S7A). Therefore, we conclude that RDR6- and DCL3-RdDM are the major activated pathways upon TE transcriptional activation and these pathways preferentially act on TEs transcribed into mRNAs. As a consequence of this shift in RdDM pathways, AGO1 indirectly contributes to the CHH methylation of more TEs (we find four–fold) in the TE-active context due to its role in the production of 21–22 nt siRNAs (Fig. 2e) [15].

In addition to the number of TE targets, we quantified the amount of CHH methylation that each RdDM pathway contributes to their respective targets. We find that in the TE-active context, when all three RdDM mechanisms are active, Pol IV-RdDM is the most efficient and causes the highest level of CHH methylation, while RDR6-RdDM and DCL3-RdDM cause less overall CHH methylation of their targets (Additional file 5: Figure S4D). The higher efficiency of Pol IV-RdDM may be due to the specialization of this pathway and its components away from post-transcriptional silencing to specifically target RdDM.

### Pol II-dependent DCL3-RdDM defines a new mechanism targeting TEs

**Fig. 3:**
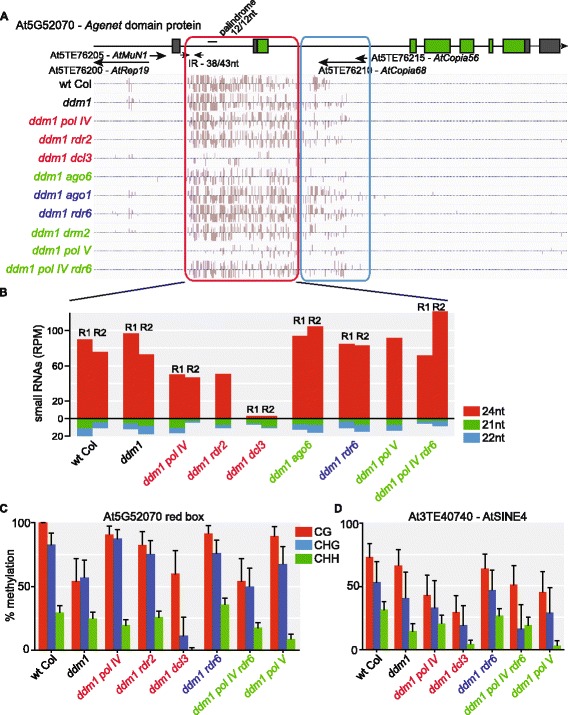
Single-locus characterization of the DCL3-RdDM pathway. **a** CHH methylation of the *AtCopia68* family fragmented TE At5TE76210 (*blue box*), which is located in the second intron of the *Agenet* domain gene At5G52070. The CHH methylation pathway targeting this TE fragment is additionally responsible for the CHH methylation of the adjacent genic introns and exon (*red box*), which contain small inverted repeat (IR) and palindromic sequences. **b** Quantification of perfectly and uniquely mapping small RNAs generated from the single copy genic region from part A (*red box*) that is targeted by DCL3-RdDM. **c** Single-locus bisulfite sequencing of biological replicate samples for the region of the At5G52070 gene in the *red box* in (**a**). **d** Single-locus bisulfite sequencing of biological replicate samples for the DCL3-RdDM target TE At3TE40740 (AtSINE4). *Error bars* in (**c**) and (**d**) indicate the 95 % confidence interval. *Coloring* of genotype labels is the same as in Fig. 2

In our analysis of TE CHH methylation patterns, we identified a category of TEs that loses methylation in *dcl3*, but not in *pol IV* or *rdr2* mutants (Fig. 2e). In the canonical Pol IV-RdDM pathway, Pol IV/RDR2-derived dsRNAs are processed into 23–24 nt siRNAs by DCL3 (reviewed in [2]). To characterize the Pol IV/RDR2-independent mechanism of DCL3-RdDM, we investigated the *AtCopia68* long terminal repeat (LTR) retrotransposon fragment At5TE76210, which is located within an intron of the *Agenet* domain gene At5g52070. We found that CHH methylation of this TE is present in *ddm1*, but lost in the *ddm1 dcl3*, *ddm1 ago6*, *ddm1 drm2*, and *ddm1 pol V* double mutants (Fig. 3a, blue box). Importantly, the CHH methylation is present in *ddm1 pol IV* and *ddm1 rdr2* mutants at a comparable level as the *ddm1* single mutant, demonstrating that the CHH methylation at this TE is not dependent on Pol IV/RDR2. The DCL3-RdDM mechanism requires Pol V, which acts downstream of siRNA production [37, 38]. Therefore, the downstream chromatin-bound portion of the DCL3-RdDM pathway acts similar to RDR6- and Pol IV-RdDM to target Pol V scaffolding transcripts with AGO-bound siRNAs, while it is only the upstream siRNA-producing portion of the pathway that differs. This DCL3-RdDM mechanism is responsible for the methylation of few TEs in the TE-silent context, but plays a larger role in CHH methylation of TEs in the *ddm1* TE-active context (Fig. 2e) and an even greater role on transcriptionally competent TEs (Additional file 8: Figure S7A), again demonstrating that this mechanism is likely dependent on Pol II transcription of its target loci.

We next aimed to characterize the siRNAs that target the DCL3-RdDM pathway. This is complicated by the fact that DCL3-RdDM targeted TEs generally have low siRNA mappability (0.78, while 1.0 equals all siRNAs map uniquely; see Additional file 2: Methods for explanation of mappability calculation) and this complicates the analysis of exactly which siRNAs are produced from these loci. We chose in Fig. 3a to investigate At5TE76210 because the methylation of the TE extends beyond the TE boundary into the single-copy sequence of the At5g52070 gene (Fig. 3a, red box). Therefore, we could unambiguously map siRNAs to this region of the genome and determine which siRNAs are driving its RdDM. We find that 24 nt siRNAs are abundantly produced from this genic region in both wt Col and *ddm1*, and the majority of these 24 nt siRNAs are not dependent on Pol IV or RDR2 in the *ddm1* TE-active context (Fig. 3b). This continued production of 24 nt siRNAs in *ddm1 pol IV* or *ddm1 rdr2* mutants correlates with the continued targeting of this region by RdDM in these mutants (Fig. 3a, red box). The 24 nt siRNAs and RdDM of this region are only dependent on DCL3 (Fig. 3a–c) and thus this represents a mechanism of Pol IV/RDR2-independent production of 24 nt siRNAs via DCL3, which can target RdDM. The production of these 24 nt siRNAs is independent of RDR2 and RDR6 and therefore this represents a distinct mechanism of 24 nt siRNA production and RdDM from the previously described RDR2-dependent or RDR6-dependent mechanisms [39, 40]. In addition, we used biological replicates and single-locus bisulfite sequencing to verify the activity of the DCL3-RdDM pathway at the gene At5g52070 and a distinct TE (At3TE40740 – AtSINE4) in the *ddm1* TE-active context (Fig. 3c, d), validating our MethylC-seq data analysis and confirming that the DCL3-RdDM mechanism is not an informatic outlier, but rather a distinct pathway that regulates multiple TEs.

### TE length is a key determinant for regulation by each type of RdDM

**Fig. 4:**
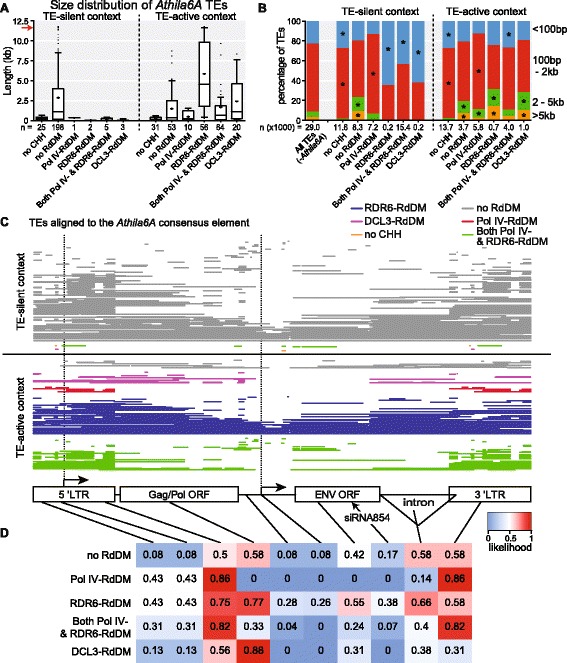
TE size correlates with RdDM pathway. **a** Size distribution of *Athila6A* LTR retrotransposons categorized by their CHH methylation pathway. The *red arrow* is the size of the *Athila6A* autonomous consensus element. *Box plot whiskers* represent 10th–90th percentile while the mean is shown as a plus sign. n = number of TEs in each group. **b** Size categorization of all Arabidopsis TEs (excluding *Athila6A* elements). *Asterisks* represent statistical significance of *p* < 0.001 using a Chi-squared test of homogeneity followed by a multiple comparison test for unequal sample sizes. **c** Individual *Athila6A* element alignment with the annotated consensus sequence (*cartoon at bottom*). Each *horizontal bar* is one element (144 analyzed in each context), where *gaps in the bar* are internal deletions or regions that do not match the consensus element. Bars are *color-coded* for the category of CHH methylation in either the TE-silent (*top*) or TE-active (*bottom*) context. **d** Likelihood *heat map* of each of the landmarks on the consensus element present in an *Athila6A* element for each specific CHH methylation pathway. The data refer to the TE-active context only

To determine how individual TEs are selected to go through different RdDM types, we analyzed individual elements from the *Athila6A* subfamily of *gypsy* LTR retrotransposons, which are strong targets of RDR6-RdDM in the TE-active *ddm1* context (Additional file 3: Figure S2A, C) [12, 16]. The majority (we find 84.6 %) of *Athila6A* elements are not targeted by RdDM in the TE-silent context and the rest of *Athila6A* TEs are smaller than 2.0 kb (the full-length *Athila6A* consensus element is 11.6 kb) (Fig. 4a). This demonstrates that the TE fragments which are too small to encode all of their own proteins (and thus by definition are non-autonomous) are either targets of RdDM or do not have detectable levels of CHH methylation, while the large potentially full-length elements are maintained in a silenced state by CMT2-based maintenance of methylation [16] and not RdDM (Fig. 4a). When transcriptionally activated, more *Athila6A* elements are targeted by RdDM (62.7 %). Although each RdDM mechanism targets some short TE fragments, we find the median size of the *Athila6A* TE that Pol IV-RdDM targets is a short 219 bp fragment, the median size that DCL3-RdDM targets is an intermediate sized 1.1 kb, while the median size that RDR6-RdDM targets is 4.5 kb (Fig. 4a). These data suggest that different RdDM mechanisms exist for long, intermediate, and short TEs.

To investigate whether the trend of long TEs specifically targeted by RDR6-RdDM is maintained genome-wide, we categorized all TEs (without *Athila6A*) by length. We find that almost all TEs with no detectable CHH methylation are small (under 2.0 kb), while most large TEs (>5 kb) undergo maintenance CHH methylation independent of RdDM in the TE-silent context (Fig. 4b). Importantly, for large TEs there is a genome-wide increase in their targeting by each type of RdDM in the TE-active context: the medium and long TEs (>2.0 kb) are statistically over-represented in the RDR6-RdDM and DCL3-RdDM categories (compared to the total genome TE size distribution) (Fig. 4b). Therefore, we conclude that when expressed, long TEs are preferentially targeted by the RDR6-RdDM and DCL3-RdDM pathways. In addition, we investigated whether TE type, proximity to a gene, position on the chromosome, or copy number correlates with RdDM type (Additional file 9: Figure S8). In contrast to TE size, we found that these other factors do not account for the switch from maintenance methylation in the TE-silent context to RdDM in the TE-active context. We did observe trends such as that the TEs without CHH methylation are typically small, high copy, and on the chromosome arms very close to genes, and that the TEs with CHH methylation that are not targeted by RdDM in the TE-silent context (and therefore undergo CMT2-based maintenance methylation) are primarily centromeric/pericentromeric and are far from genes. Pol IV-RdDM preferentially targets chromosome arm TEs near genes, which correlates with previous data [7, 19, 22]. DCL3- and RDR6-RdDM preferentially target TEs far from genes in the centromere/pericentromere and favor the long LTR retrotransposons that are found at these regions and dominate large plant genomes.

We next aimed to correlate the genetic structure of individual TEs with their specific RdDM regulatory pathway. Most Arabidopsis TEs lack a detailed annotation based on structure and RNA expression data. We characterized the well-studied *Athila6A* consensus TE to define the transcriptional start sites, open reading frames (ORFs), and intron (data summarized in Fig. 4c). We aligned individual *Athila6A* TEs to the full-length annotated consensus element and categorized them by the CHH methylation pathway in either the TE-silent or TE-active context (Fig. 4c). As in Fig. 4a, we find that in the TE-silent context very few *Athila6A* elements are targeted by RdDM and these are only small TE fragments. In the TE-active context, the *Athila6A* elements are spread among the various RdDM categories. Importantly, we find that all full-length elements are specifically targeted by the RDR6-RdDM pathway (Fig. 4c). In addition, Pol IV-RdDM only targets small LTR fragments, while other fragmented or larger internally deleted elements are spread among all of the other CHH methylation pathways, including RDR6-RdDM. Of the known *Athila6A* features required for autonomous transposition (production of all the necessary proteins required for self-mobilization or mobilization of non-autonomous elements), including LTRs, transcriptional start sites, and ORFs, we find the probability of an element to encode this feature correlates with its CHH methylation pathway in the TE-active context (Fig. 4d). For example, elements targeted by Pol IV-RdDM never (in our dataset) contain any of the *Athila6A* internal coding region, while elements targeted by DCL3-RdDM always have an internal deletion of the ENV ORF promoter. Of interest, nearly all elements that retain the ENV ORF promoter are targeted for RDR6-RdDM in our dataset, suggesting that this structure is directing RDR6-RdDM activity on these transcripts. From these data we determine that RDR6-RdDM does not specifically act only on full-length elements, but all full-length and structurally autonomous *Athila6A* elements are targeted specifically by RDR6-RdDM. Therefore, which particular small RNA silencing pathway regulates each TE is influenced by the TE's genetic structure. 

### Structurally autonomous TEs are preferentially targeted by RDR6-RdDM

**Fig. 5:**
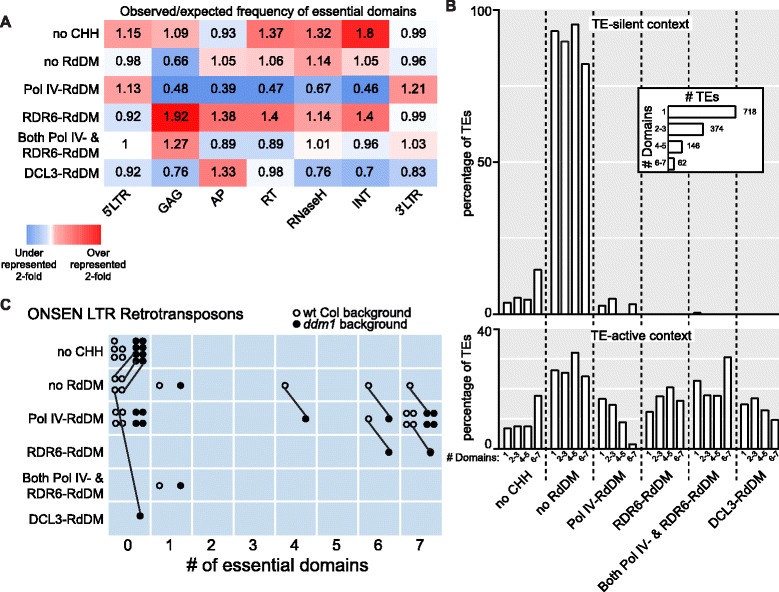
Expression-dependent forms of RdDM regulate structurally autonomous and near-complete active LTR retrotransposons. **a** Ratio of observed/expected frequency *heat map* of each of the seven domains essential for LTR retrotransposition for each specific CHH methylation pathway. These data refer to the TE-active context only. **b** The RdDM categorization of LTR retrotransposons (excluding ONSEN elements) based on the number of domains essential for LTR retrotransposition listed in (a). *Inset graph* displays the total number of LTR retrotransposons in each category. **c** The change in RdDM categorization for individual ONSEN family TEs from the wt Col to *ddm1* backgrounds. Each ONSEN element is represented twice, once as an *open dot* (wt Col background) and again as a *closed dot* (*ddm1* background). *Lines* connecting an open dot to a closed dot represent shifts of individual ONSEN elements from one CHH methylation category to another upon switching to the *ddm1* background

To determine if the trend of full-length *Athila6A* elements preferentially targeted by RDR6-RdDM is consistent with all LTR retrotransposons, we profiled each LTR retrotransposon for the presence or absence of seven domains essential for retrotransposition: 5’ LTR, GAG capsid protein, AP protease, RT, RNaseH, INT protein, and 3′ LTR. We determined the probability of each RdDM pathway to target a TE with these domains in the TE-active context (Fig. 5a). We found that elements regulated by RDR6-RdDM generally possess all of the internal protein-coding regions, while particular TEs with the GAG protein domain are more often targeted by RDR6-RdDM. Similar to our finding with *Athila6A*, we find that Pol IV-RdDM targets TEs that have a low probability of containing any of the internal retrotransposition-essential domains and elements targeted by DCL3-RdDM have a reduced probability of containing the protein-coding regions GAG, RNaseH, or INT (Fig. 5a).

We next aimed to determine if LTR retrotransposons with all of the domains required for retrotransposition are targeted by one RdDM type. Few LTR retrotransposons have all seven of the domains defined in Fig. 5a, so we clustered the TEs into categories of 1, 2–3, 4–5, and 6–7 domains (inset Fig. 5b). In the TE-silent context, most of the TEs with 6–7 domains are not targeted by RdDM and rather are subject to maintenance methylation. In the TE-active context, Pol IV-RdDM alone acts on few 6–7 domain elements, while the RDR6-RdDM, DCL3-RdDM and co-regulated Pol IV- and RDR6-RdDM categories function on the majority of the elements with all the necessary domains required for retrotransposition. Of note, a trend exists where the higher number of retrotransposition-essential domains an LTR-retrotransposon has, the less likely that TE is to be targeted by Pol IV-RdDM in the TE-active context. These trends remain consistent, but are not further enriched, when the subset of transcriptionally competent TEs is interrogated (Additional file 8: Figure S7B).

One outlier TE family to the trends observed in Fig. 5b is ONSEN, a heat-activated *Copia* LTR retrotransposon (*Copia78*) [14, 41]. For ONSEN, most elements with 6–7 essential retrotransposition domains are targeted by Pol IV-RdDM in the wt Col background and they remain targeted by Pol IV-RdDM in the *ddm1* background (Fig. 5c). Two ONSEN elements behave like most other LTR retrotransposons and in the *ddm1* background switch to being regulated by RDR6-RdDM, but ONSEN is unusual in the fact that many near-complete elements remain Pol IV-RdDM targets in the *ddm1* background. Why the ONSEN family behaves differently than other LTR retrotransposons is unclear, but it is likely due to the fact that ONSEN is not transcriptionally activated in *ddm1* mutants that have not been heat-activated [14].

### Full-length TEs are preferentially targeted for mRNA cleavage and secondary siRNA production, driving RDR6-RdDM

**Fig. 6:**
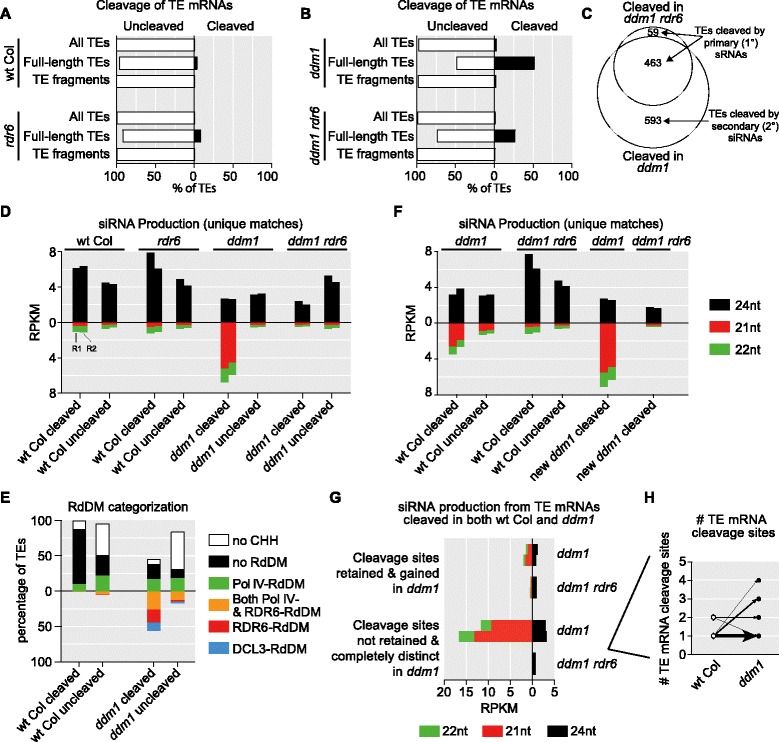
Full-length TEs are preferentially cleaved and produce secondary siRNAs, driving RDR6-RdDM. **a** Percentage of TEs with cleaved mRNAs in the TE-silent context. **b** Percentage of TEs with cleaved mRNAs in the TE-active context. In (a) and (b) *rdr6* mutants are used to determine which cleavage events are due to primary small RNAs (not RDR6 dependent) or RDR6-dependent secondary siRNAs. **c** *Venn diagram* of cleaved TE mRNAs in the TE-active context and their dependence on RDR6-generated secondary siRNAs. **d** siRNA production from small RNA-cleaved and uncleaved TE mRNAs determined in parts (a–c). *Labels above bars* indicate the assayed genotype of small RNA alignment. **e** RdDM pathway categorization of the TE categories shown in (d). **f** siRNA production in the TE-active context. Small RNA-cleaved and uncleaved TE mRNAs in the TE-silent context are the same as in (d). “New *ddm1* cleaved” refers to TE mRNAs that are cleaved in the TE-active context but were not cleaved in the TE-silent context. **g** TE-active context siRNA production from TE mRNAs with new cleavage sites in the TE-active context compared to the TE-silent context. “Cleavage sites retained & gained in *ddm1*” refers to TE mRNAs with new cleavage sites plus the retention of the same TE mRNA cleavage sites from the TE-silent context. “Cleavage sites not retained & completely distinct in *ddm1*” refers to TE mRNAs with new cleavage sites without retention of the same TE mRNA cleavage sites from the TE-silent context. **h** Of the “Cleavage sites not retained & completely distinct in *ddm1*” TEs from (g), the number of cleavage sites on the same TE mRNA in the TE-silent and TE-active contexts. Weight of the *arrow* is determined by how many TE mRNAs transition from 1–2 cleavage sites in the TE-silent context to other numbers of cleavage sites in the TE-active context. The majority of TE mRNAs have one cleavage site in the TE-silent context and a different one cleavage site in the TE-active context

Key remaining questions are how and why long autonomous TEs are preferentially targeted by RDR6-RdDM. To investigate this preference, we measured the length of each TE compared to its autonomous consensus element and categorized individual TEs as full-length or TE fragments (see “Methods”). Creasy et al. found that TE mRNAs are targeted for initial cleavage by microRNA-like primary (not dependent on a RDR protein) small RNAs produced elsewhere in the genome [29]. They also demonstrated that this cleavage is responsible for initiating RDR6-dependent RNAi and production of 21–22 nt secondary siRNAs [29] and these secondary siRNAs drive RDR6-RdDM [12, 16]. We used this mRNA cleavage data from the same tissue of wt Col TE-silent and *ddm1* TE-active context genotypes to determine if the preference of full-length TEs to enter RDR6-RdDM is dictated on the mRNA-cleavage level. Therefore, we compared the percentage of full-length and fragmented TEs that are cleaved by primary small RNAs. As expected, few TE mRNAs are cleaved in the TE-silent context (Fig. 6a), since not many full-length TEs are expressed in wt Col (Additional file 1: Figure S1). In the TE-active context more TE mRNAs are expressed and are cleaved and we detected that the cleaved TE mRNAs are mostly from full-length TEs (Fig. 6b). This trend holds true for all TE types and is not specific to LTR retrotransposons (Additional file 10: Figure S9A, B). Additionally, by comparing the cleavage data from *rdr6* and *ddm1 rdr6* mutants, we were able to categorize TEs specifically targeted by primary small RNAs (not dependent on RDR6) or secondary siRNAs (dependent on RDR6) in both the TE-silent and TE-active backgrounds (Fig. 6c). We find that the small amount of detectable TE mRNA cleavage in the TE-silent context is occurring via primary small RNAs and in the TE-active context both primary and secondary small RNAs cause TE mRNA cleavage (Fig. 6a, b).

We next aimed to determine if the preference for full-length TE mRNA cleavage in the TE-active context results in secondary siRNA production from specifically the full-length cleaved TEs. In the TE-silent context, TEs that produce either cleaved or uncleaved mRNAs generate similar siRNA distributions, which are predominantly 24 nt (Fig. 6d), demonstrating that in the TE-silent context the small amount of TE cleavage does not lead to additional siRNA production. In the TE-active context, cleaved TE mRNAs generate RDR6-dependent 21–22 nt siRNAs, while as expected the uncleaved TE mRNAs do not (Fig. 6d). In addition, it is only the TEs with cleaved mRNAs in the TE-active context that are subject to RDR6-RdDM without Pol IV-RdDM compensation (Fig. 6e). Therefore, the reason most full-length structurally autonomous TEs are targeted by RDR6-RdDM in the TE-active context is: (1) full-length TEs are preferentially cleaved by primary small RNAs (Fig. 6a, b); (2) only cleaved TE mRNAs in the TE-active context make RDR6-dependent secondary 21–22 nt siRNAs (Fig. 6d); (3) only secondary 21–22 nt siRNA production drives RDR6-RdDM [16].

### New primary sRNAs that accumulate in the TE-active context direct TE mRNA cleavage and drive RDR6-RdDM specificity

Since cleavage of full-length TE mRNAs can be detected in both the TE-silent and TE-active contexts (Fig. 6a, b), we wondered why RDR6-RdDM is only activated in the TE-active context. We therefore aimed to determine if secondary siRNAs generated in the TE-active context are from: (1) the same TE mRNAs cleaved in both the TE-silent and TE-active context; or (2) from cleavage of new TE mRNAs that were not expressed or uncleaved in the TE-silent context. We found that there are new TEs subject to mRNA cleavage in the TE-active context and these mRNAs produce 21–22 nt secondary siRNAs (new *ddm1* cleaved, Fig. 6f). Additionally, we found that for the TE mRNAs that are cleaved in the TE-silent context (which do not produce secondary siRNAs), in the TE-active context these exact same TE mRNAs produce 21–22 nt secondary siRNAs (wt Col cleaved, Fig. 6f). Therefore, why does the same cleaved TE mRNA not produce secondary siRNAs in the TE-silent context while it efficiently produces secondary siRNAs in the TE-active context? We generated four hypotheses: (1) increased mRNA expression and hence increased mRNA cleavage at the same site in the *ddm1* TE-active context drives secondary siRNA production; (2) equal numbers of new TE mRNA primary cleavage sites accumulate in the TE-active context, resulting in secondary siRNA production; (3) cleavage by multiple primary sRNAs drives secondary siRNA production only in the TE-active context [42]; and (4) the primary sRNAs directing TE mRNA cleavage are 21 nt in the TE-silent context, but are 22 nt in the TE-active context, a size shift that is known to induce secondary siRNA production [29, 43]. We individually tested these hypotheses (Fig. 6g, h, Additional file 10: Figure S9C, D) and found that TE mRNA cleavage occurs at new distinct sites within the TE mRNAs, driving RDR6-function and secondary siRNA production (Fig. 6g), while the size of the siRNA, level of TE mRNA, and the number of cleavage sites did not contribute (Fig. 6h, Additional file 10: Figure S9C, D). We observed that many TE mRNAs are cleaved once in the TE-silent context and once in the TE-active context, but the change in the primary sRNA and/or cleavage site results in secondary siRNAs production only in the TE-active context (Fig. 6g, h). Thus, new primary small RNAs with the same size distribution appear in the TE-active context and cleave the same TE mRNAs at new positions and are responsible for the TE 21–22 nt secondary siRNA production that drives RDR6-RdDM of full-length elements specifically in the TE-active context.

## Discussion

### DCL3-RdDM defines a new pathway of TE silencing

By using MethylC-seq and single-locus bisulfite sequencing, we identified and confirmed a novel RdDM pathway that acts via 24 nt siRNAs produced not from Pol IV transcripts, but rather presumably from Pol II transcripts cleaved by DCL3. This pathway is distinct from the Pol II-RDR6-DCL3 or Dicer-independent pathways previously described [35, 40]. On the whole-genome level, we found that this pathway acts on transcriptionally active TEs that are typically long but internally deleted versions of the full-length autonomous element. For *Athila6A* sub-family TEs, all individual TE targets of DCL3-RdDM are missing the internal promoter region (Fig. 4c, d), suggesting that the structure of the TE critically drives individual elements into this pathway. We focused on a single-locus example to define the precise protein requirements for siRNA production and targeting of DCL3-RdDM and we find that the upstream portion of the pathway is distinct from Pol IV-RdDM, while the downstream portion of the pathway (involving Pol V, AGO6, and DRM2) is conserved. We find that the DCL3-RdDM pathway is independent of RDR2 and RDR6, but it is unknown if this pathway functions completely independently of RNA-dependent RNA polymerization (RDR1, 3, 4, and 5 are untested). If RDR-independent, this pathway may function on only intra-molecular dsRNA generated from fold-back RNA hairpins [44]. The At5g52070 methylated region contains a short inverted repeat and a short palindrome sequence (Fig. 3a), but it is unknown if these features or this locus produces a fold-back dsRNA substrate for DCL3 processing. We investigated whether DCL3-RdDM target TEs are associated with palindromes or inverted repeats genome-wide; however, we did not detect a correlation (data not shown). In addition, we do not see an increase in 21–22 nt siRNA production in *dcl3* mutants at the DCL3-RdDM target At5g52070 (Fig. 3b), suggesting that multiple DCL proteins are not competing for the same dsRNA substrate. These RNA substrates are likely produced by Pol II, as we detect more DCL3-RdDM in the TE-active context, DCL3-RdDM is enriched for targets in the transcriptionally competent TE subset, and the 24 nt siRNAs driving DCL3-RdDM are not dependent on Pol IV or Pol V (Fig. 3b). However, key insights, such as the developmental stage that DCL3-RdDM is active and whether DCL3-RdDM functions in the initiation and/or re-establishment of TE silencing, remain unknown.

### Diversity of RdDM mechanisms

TEs are the genome-wide target of RdDM mechanisms [19, 45]. In this study we investigated RdDM mechanisms in both the wt TE-silent context, as well as a *ddm1* mutant context with genome-wide transcriptionally active TEs. The *ddm1* mutation prohibits the formation of the transcriptionally silenced state and hence the cell is in a perpetual cycle of attempted targeting of re-silencing via RdDM. This uncovered an unappreciated diversity in RdDM mechanisms that function on TE targets in the TE-active context. It is now clear in the RdDM field that many biogenesis mechanisms can produce small RNAs that are loaded into AGO proteins to participate in RdDM, including Pol IV/RDR2 24 nt siRNAs (Pol IV-RdDM), Pol IV-independent 24 nt siRNAs (DCL3-RdDM), 21–22 nt siRNAs via RDR6-RdDM, and Dicer-independent siRNAs. We found that the specific pathway targeting each TE is largely defined by the TE’s structure. Our data demonstrate that the cell utilizes a number of distinct pathways to direct DNA methylation to active TEs; however, all of these mechanisms converge on one downstream chromatin modifying complex that includes Pol V and DRM2.

Pol IV transcribes silent TEs [5] and thus Pol IV-RdDM functions in the TE-silent context to maintain DNA methylation at particular short TEs near genes, which may require constant re-targeting to maintain the boundary between the heterochromatic TE fragment and the euchromatic neighboring gene [8] (reviewed in [46]). In the TE-silent context, RDR6-RdDM and DCL3-RdDM do not function on many TEs, presumably due to the lack of Pol II-derived transcripts. In contrast, in the TE-active context, DCL3-RdDM and RDR6-RdDM function on 20 % of assayed TEs (and 40 % of transcriptionally competent TEs). In particular, DCL3- and RDR6-RdDM target long TEs which are farther from genes (within the centromeres/pericentromeres); however, DCL3-RdDM targets TEs with internal deletions, while full-length elements are targeted specifically by RDR6-RdDM (at least for the well-annotated *Athila6A* family). This phenomenon is likely conserved in other plants, as in maize an active autonomous TE is regulated by RDR6-RdDM [47]. However, there are notable exceptions to these general trends, demonstrating that cases exist where TE family-based regulatory dynamics can outweigh the TE size/structure-based regulation shown in Figs. 4 and 5. For example, all RdDM pathways regulate many small fragmented TEs (including RDR6-RdDM), and many full-length ONSEN family TEs are regulated by Pol IV-RdDM even in *ddm1* mutants. How and when TE family-based regulation outweighs other trends in genome-wide regulation of TEs remains a key question to be addressed.

Within the specific regulation of individual TE copies, we found that RdDM mechanisms can compensate for each other. In the TE-active context, when Pol IV is mutated many TEs display CHH methylation that is now dependent on RDR6 and vice versa Pol IV-RdDM compensation is detected in *rdr6* mutants. However, this compensation effect was not detected unless both Pol IV and RDR6 were mutated at the same time. This compensation may be due to a competition between Pol IV and Pol II for DNA substrates [37, 48]. Thus, only when Pol IV is mutated would Pol II transcribe these TEs into substrates for RDR6-RdDM.

### Specificity of full-length TEs for RDR6-RdDM

**Fig. 7:**
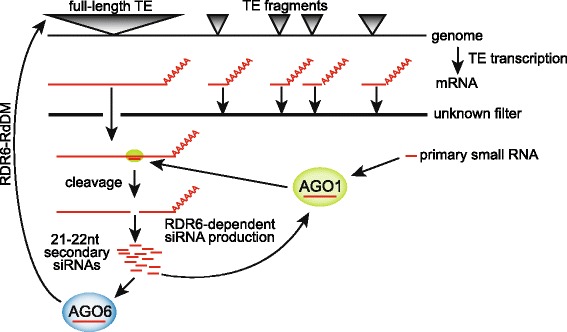
*Model* of full-length TE specificity for RDR6-RdDM. Full-length TE mRNAs are specifically cleaved and degraded into secondary 21–22 nt siRNAs, driving the specificity of RDR6-RdDM. An unknown filter blocks the mRNA cleavage, RNAi, and secondary siRNA production of mRNAs from fragmented TEs

Because many active full-length structurally autonomous TEs are regulated by RDR6-RdDM, a major question was what drives this specificity. Primary small RNAs are produced either from TEs themselves or elsewhere in the genome by a mechanism similar to microRNA production [29]. Our analysis suggests a model whereby full-length TEs are more likely regulated by RDR6-RdDM due to the specificity of full-length TE mRNAs for cleavage by primary sRNAs. Full-length TE mRNAs may circumvent RNA surveillance mechanisms that target short fragmented RNAs, allowing only the full-length high-quality TE mRNAs to survive to the point where they can be targeted by primary sRNAs (see model in Fig. 7). The nature of the filter responsible for shielding fragmented TE mRNAs from siRNA cleavage is currently unknown (Fig. 7); however, it may be as simple as the nuclear envelope acting as a mRNA quality control filter, allowing full-length TE mRNAs export into the cytoplasm (for cleavage by sRNAs), while the fragmented TE mRNAs are not exported and instead degraded by alternative mechanisms. XRN endonuclease and exosome RNA degradation competes with RNAi [49] and the mRNAs produced from TE fragments may be degraded by these mechanisms rather than be targeted by a primary sRNAs and enter into RNAi. In the TE-active context, once cleaved, the TE mRNAs are targeted by RDR6 and abundant secondary siRNAs are produced. These secondary siRNAs promote additional rounds of RNAi of TE mRNAs (via AGO1), as well as drive RDR6-RdDM (via AGO6).

A major question is why some mRNA cleavage events from primary sRNAs generate secondary siRNAs while others do not. For example, some TE mRNAs are cleaved by primary sRNAs in both the TE-silent and TE-active contexts; however, secondary siRNAs are only produced in the TE-active context. We determined that these TE mRNAs are cleaved at new positions by new primary small RNAs in the TE-active context and this is responsible for their secondary siRNA production via RDR6. One hypothesis in the field is that the size of the primary small RNA (21 vs. 22 nt) drives the distinction between secondary siRNA production [43]; however, we did not detect any size shift in the small RNAs (Fig. 6g, h and Additional file 10: Figure S9D). Another hypothesis is that multiple primary cleavage events on the same mRNA triggers secondary siRNA production [42]; however, we did not detect a correlation between multiple cleavage sites and secondary siRNA production. Therefore, how RDR6 is recruited to some cleaved transcripts to generate secondary siRNAs remains a key open question in the field.

## Conclusions

The tight transcriptional silencing of TEs in the reference strain of Arabidopsis has produced a general lack of appreciation for the diversity of RdDM mechanisms. We focused on TE silencing mechanisms in both the TE-silent and TE-active contexts and conclude that multiple small RNA-generating mechanisms can target RdDM when TEs are transcriptionally active. This includes the DCL3-RdDM pathway, which processes 24 nt siRNAs for RdDM independent of Pol IV. We found that TE structure and length are key determinants for RdDM pathway specificity and in particular RDR6-RdDM targets many of the full-length and structurally autonomous TEs in the genome. The targeting preference of RDR6-RdDM for full-length autonomous TEs is generated from the specificity of full-length TE mRNAs to be cleaved by primary small RNAs and therefore this RNA cleavage specificity drives the initiation of long-term epigenetic repression of TE mobility.

## Methods

### Plant material

All plants used in this study are in the Col ecotype background of *Arabidopsis thaliana*. Plants were grown in long-day (18-h light) conditions at 22 °C and stage 1–12 inflorescence tissue (staging as in [50]) was used for all experiments and sequencing. The alleles of the mutants are shown in Additional file 11: Table S1.

### MethylC-seq

DNA was isolated using fractional precipitation followed by phenol-chloroform extraction and *RNase A* treatment. A total of 1 ug of DNA was used to prepare libraries as previously described [51]. Single-end Illumina sequencing of 150 bp was performed at the University of Georgia Genomics Facility using an Illumina NextSeq500 instrument.

### Mapping of MethylC-seq data

Sequencing reads were trimmed for adapters, preprocessed to remove low quality reads, and aligned to the *A. thaliana* TAIR10 reference genome as previously described [52, 53]. Two strategies were performed to map these data: (1) uniquely mapping: any reads that mapped to more than one location were discarded *(-m 1*); and (2) multi-mapping: reads that mapped to multiple locations were retained (*-a*). Both strategies do not allow PCR duplicated reads. We calculated the average TE mappability from the uniquely mapping methylation dataset and found 67.5 % of TEs are perfectly (100 %) mappable, whereas another 32.1 % are semi-mappable, and 0.36 % are not mappable (Additional file 2: Results and Methods and Additional file 3: Figure S2). Therefore, if the read length is long enough (in this study, the read size is 150 bp) and the repetitive fraction of a genome is small/simple/divergent enough (the Arabidopsis genome), then using the unique mapping strategy can provide sufficient coverage and mappability to interrogate TE methylation dynamics, with the added benefit of determining the unique methylation states of individual TE copies. All data analyses shown in this study (excluding Additional file 3: Figure S2) are produced from the uniquely mapping strategy. Sequencing and mapping statistics of our MethylC-seq data is shown in Additional file 11: Table S1.

### DMR identification

DMRs were identified between all datasets as previously described [54]. The maximum physical distance to combine two differentially methylated sites (DMSs) was set to 250 bp. DMRs with at least four DMSs were reported and used for subsequent analyses.

### Methylation level calculation

The weighted methylation level of genomic features (TE, gene, or DMR) was calculated as described previously [55].

### Meta-plots

For each individual entity (TE, gene, or DMR), the average CHH methylation percentage was calculated in 100 bp windows across the length of the entity and a 2 kb neighboring region on either side. The entities are either aligned at the 5′ end or the 3′ end and the average methylation percentage for all the elements was calculated for each 100 bp window. For DMRs, there is no defined 5′ or 3′ end and hence only one edge is shown. For any given window, the variation in methylation across all elements was used to calculate the 95 % confidence interval.

### RdDM mechanism categorization

Only those TEs which are covered (at least one reported cytosine) in all genotypes were used for RdDM categorization and further analysis. For each TE, we calculated the average CHH methylation and only this specific cytosine context was used for TE categorization. TEs with less than 1 % CHH methylation in wt Col were classified into the “no CHH” category. For all the other RdDM categories, the following criteria were used for classification. Pol IV-RdDM: TEs that lose >2-fold methylation in *pol IV* and *pol V* compared to wt Col but do not lose >2-fold methylation in *rdr6* or *pol IV rdr6* compared to wt Col or *pol IV*, respectively. RDR6-RdDM: TEs that lose >2-fold CHH methylation in *rdr6* and *pol V* compared to wt Col, but not *pol IV* or *pol IV rdr6* compared to wt Col or *rdr6*, respectively. DCL3-RdDM: TEs that lose >2-fold CHH methylation in *dcl3* and *pol V* but not in *pol IV* or *rdr6* or *pol IV rdr6* double mutants. The Pol IV- and RDR6-RdDM co-regulated category: TEs that lose CHH methylation in the *pol IV rdr6* double mutant compared to wt Col but do not belong to either Pol IV- or RDR6-RdDM categories. No RdDM: TEs with greater than 1 % CHH methylation but do not lose >2-fold CHH methylation in a *pol V* mutant or could not be categorized into any of the above-mentioned RdDM categories. For the TE-active context, the corresponding *ddm1* double mutants were compared to the *ddm1* single mutant for categorization. The heat map shown in Fig. 2e was created using the average methylation for each TE in each genotype using the *heatmap.2* function of the *gplots* package in *R*. The TEs were sorted by their average CHH methylation in each RdDM category for either the TE-silent or the TE-active context.

### Small RNA data mapping

Small RNAs were isolated, sequenced, and processed as in [16]. Low quality and reads from rRNA and tRNAs were removed. *Bowtie* (version 1.1.1) [56] was used to map the sRNAs to specific regions or the whole genome. For sRNA production, only uniquely and perfectly mapping reads were considered (*bowtie* parameters: *-v 0 -m 1*). Reads per million (RPM) for each size class of mapped sRNAs was calculated by normalizing the number of raw mapped reads to the total genome-matched (non-tRNA/rRNA) 18–28 nt reads of the specific sRNA library. For reads per kilobase per million (RPKM), the RPM value was normalized to the total length of the region(s) that the sRNAs were mapped to. Heat maps in Fig. 2 were generated using the *heatmap.2* function of *gplots* package in *R*.

### TE consensus element alignments

For Fig. 4, the *Athila6A* consensus sequence from GIRI RepBase was used [57]. We aligned all the TAIR10 TEs annotated as *Athila6A* to the consensus sequence using *Blastn*. The number of TEs undergoing a specific RdDM mechanism was calculated and the fraction of those TEs having a specific *Athila6A* annotation feature is shown in the likelihood map in Fig. 4d. In Fig. 6a–c, length of each TE in the TAIR10 annotation was compared to its specific autonomous consensus element sequence from GIRI RepBase. TEs were divided into full-length (>80 % of autonomous consensus element length) and TE fragments (<20 % length).

### LTR domain annotation and analysis

To identify the TEs with one or more of the essential retrotransposition domains, the TAIR10 TE annotation was used to predict all possible LTR retrotransposon peptide fragments (all reading frames split by stop codons). The peptides were used to query in the HMM domain libraries [58] using *hmmsearch* [59]. Only hits covering more than 90 % of the reference essential retrotransposition HMM domains were considered. LTRs were predicted using *TRsearch* from *REPET* [60], which identifies LTR pairs within a single element. This identified 407 LTR pairs, which were used as a *blastn* library to identify 894 single LTRs on fragmented elements. LTRs found within the internal portion (farther than 50 bp from either end) of a TE element were discarded. The frequency of each essential domain (number of TEs with a specific domain/total number of TEs) was calculated for all TEs (expected frequency) and for TEs in each specific RdDM category (observed frequency). The ratio of observed over expected frequency is shown as a heat map in Fig. 5a.

### mRNA cleavage data analysis

Published and processed data that report the sRNA and its detected PARE signature from [29] were used to assay mRNA cleavage. Only cleavage sites with *p* value < 0.05 were considered. From this dataset, we determined if one specific TE mRNA is being cleaved at one or multiple sites.

### Identification of transcriptionally competent TEs

TEs were identified with at least one uniquely matching RPM in *ddm1* RNA-seq (GSE38286) [61], at least ten uniquely matching RPM of 21–22 nt (Pol II-derived) siRNAs in *ddm1* (GSE57191) [16], or any evidence of mRNA cleavage in *ddm1* or *ddm1 rdr6* in GSE52342 [29].

### Single-locus bisulfite sequencing

Bisulfite sequencing was done as in McCue et al. [16] with the PCR primers shown in Additional file 12: Table S2.

## Additional files

Additional file 1: Figure S1. Steady-state TE mRNA accumulation in *ddm1* and the RdDM mutant *pol V*. Full-length TEs undergo a larger shift in reactivation in *ddm1* mutants compared to *pol V* mutants. In *pol V* mutants, only a slight genome-wide activation of TEs is detected. (PDF 164 kb) Additional file 2: Results and Methods. Detailed Results, Methods, Figure Legends, and References to support the Supplemental Figures and Tables. (PDF 141 kb) Additional file 3: Figure S2. MethylC-seq has the resolution to assay RDR6-RdDM on individual TE loci. Using our unique-mapping approach and our MethylC-seq dataset, the highly repetitive TE targets of RDR6-RdDM are assayable, which allows for locus-specific analysis of TE methylation patterns. (PDF 188 kb) Additional file 4: Figure S3. Validation of MethylC-seq data using biological replicates of key genotypes. Analyses of biological replicates of key samples showing the reproducibility of major conclusions from our study. (PDF 317 kb) Additional file 5: Figure S4. Overlap in DMRs and TEs regulated by RdDM. Comparison of the relative efficacy of RdDM mechanisms in both TE-silent and TE-active contexts. (PDF 176 kb) Additional file 6: Figure S5. Genome-wide distribution of TE CG and CHG methylation. Heatmap showing CG and CHG methylation for the TEs categorized into different RdDM mechanisms based on CHH methylation. (PDF 394 kb) Additional file 7: Figure S6. Pol IV- and RDR6-RdDM compensate for each other. Evidence of RdDM compensation is observed when both Pol IV and RDR6 are mutated simultaneously in either TE-silent or TE-active context. (PDF 105 kb) Additional file 8: Figure S7. Enrichment of RDR6-RdDM and DCL3-RdDM at transcriptionally competent TEs. We find that expression-dependent forms of RdDM are enriched when investigating the transcriptionally competent subset of TEs. (PDF 403 kb) Additional file 9: Figure S8. Correlation between CHH methylation pathway and TE location, type, and copy number. Genome-wide trends exist for correlation of TE location, type, and copy number with the type of RdDM mechanism. (PDF 141 kb) Additional file 10: Figure S9. TE cleavage dynamics. Cleavage site analyses demonstrate that size of the primary small RNAs and TE mRNA accumulation level do not dictate secondary siRNA production. (PDF 153 kb) Additional file 11: Table S1. Mutant alleles and quality statistics of the MethylC-seq data produced in this study. Reads from our MethylC-seq dataset cover more than 96 % of all cytosines in the genome with a roughly 25-fold coverage. (PDF 48 kb) Additional file 12: Table S2. Primer sequences used in this report. Primer sequences for single-locus bisulfite sequencing performed in Fig. 3c, d are reported. (PDF 36 kb)
