# Supplementary figures and images for: Full-length autonomous transposable elements are preferentially targeted by expression-dependent forms of RNA-directed DNA methylation
Source: Genome Biol. 2016 Aug 9;17:170. doi: 10.1186/s13059-016-1032-y (PMC4977677; doi:10.1186/s13059-016-1032-y)

**Figure S1**

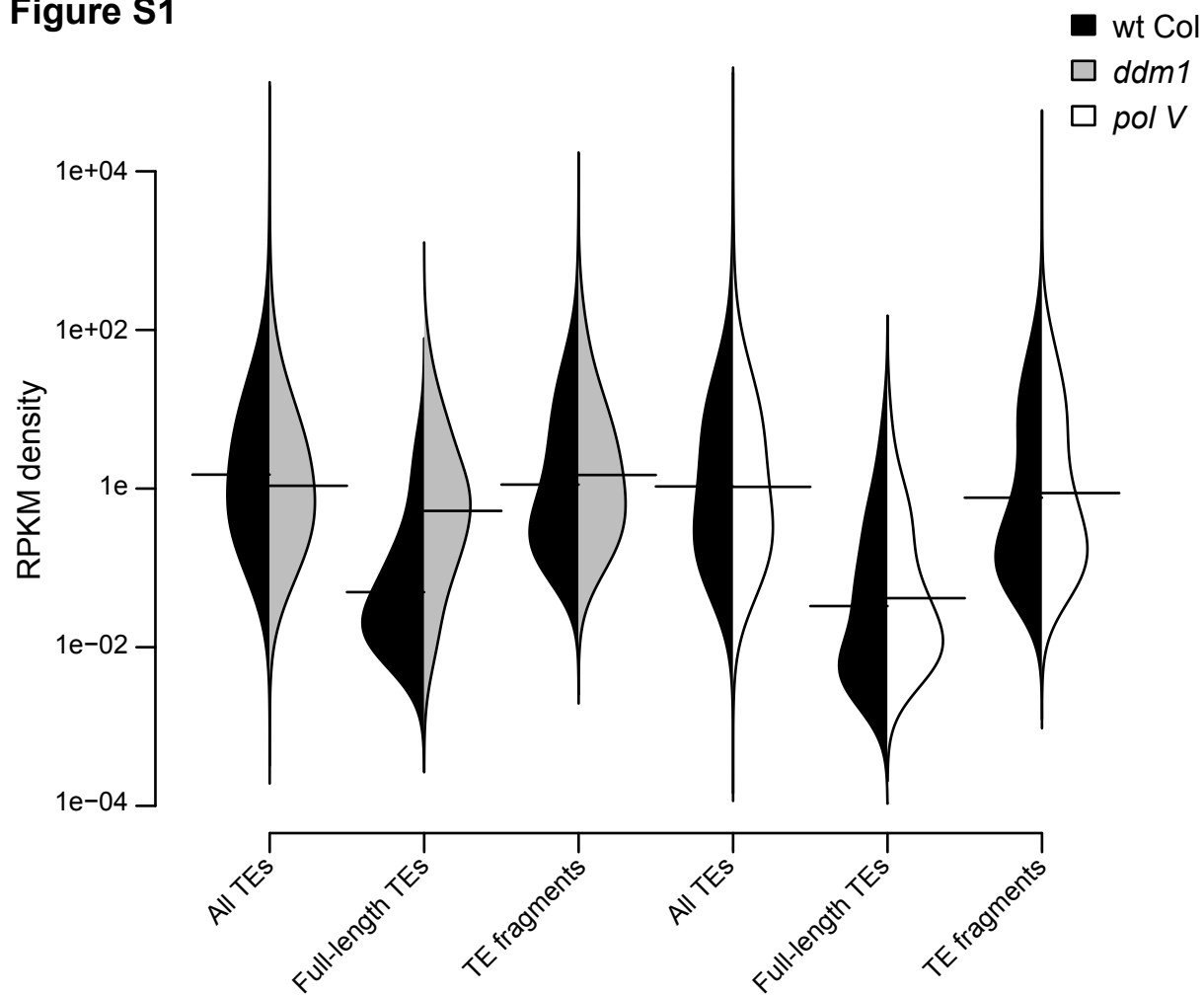

Supplement: Additional file 1: Figure S1. — Steady-state TE mRNA accumulation in ddm1 and the RdDM mutant pol V. Full-length TEs undergo a larger shift in reactivation in ddm1 mutants compared to pol V mutants. In pol V mutants, only a slight genome-wide activation of TEs is detected. (PDF 164 kb) [file 13059_2016_1032_MOESM1_ESM.pdf]

### Figure S2

A

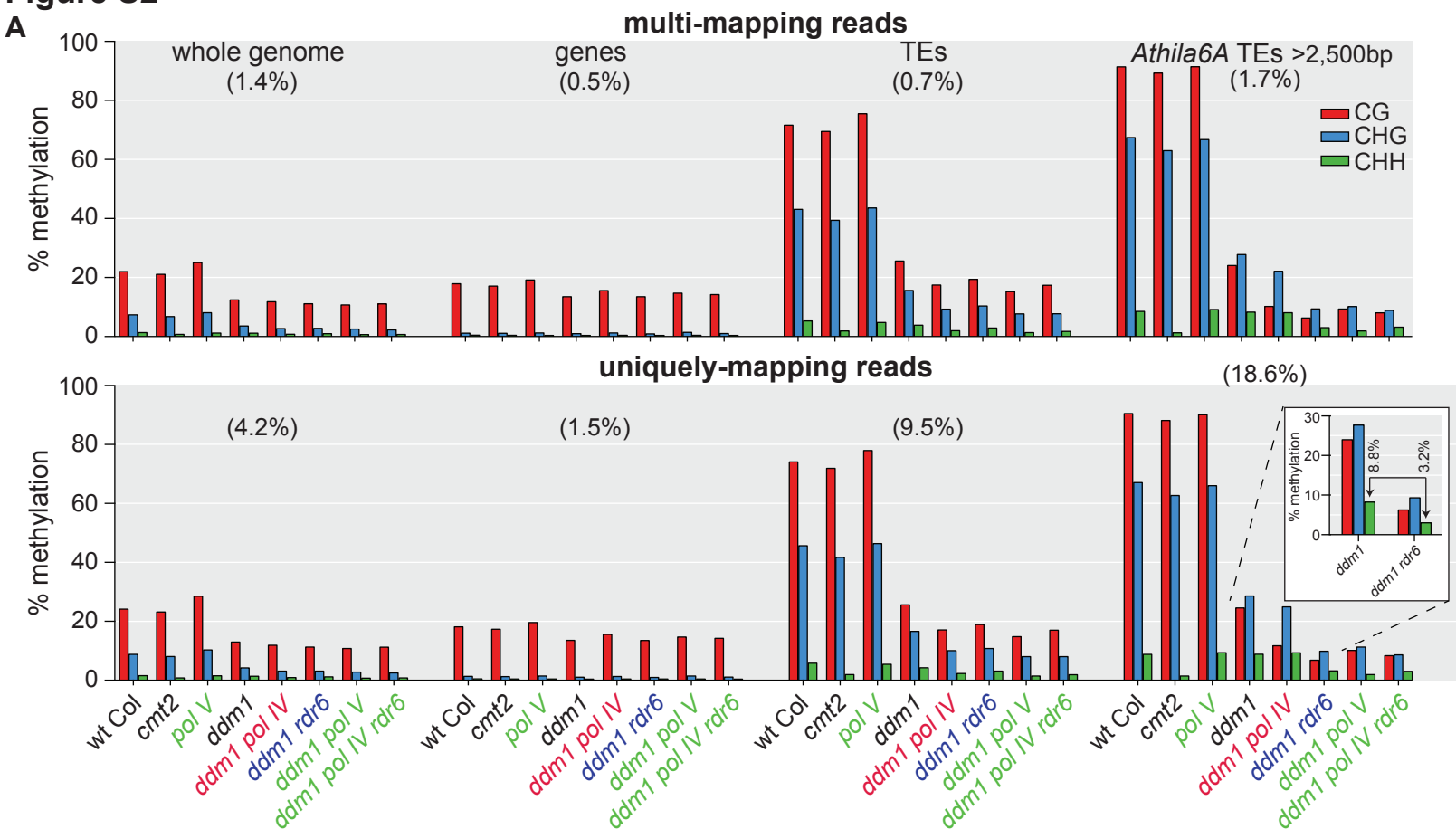

B

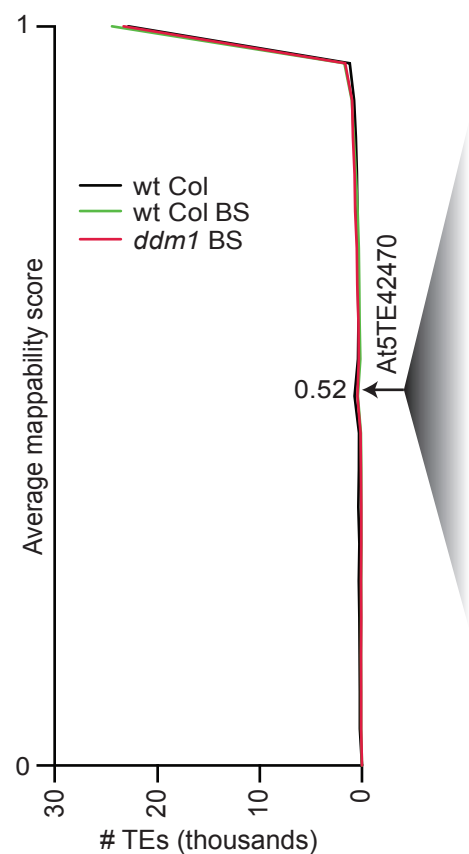

**C**

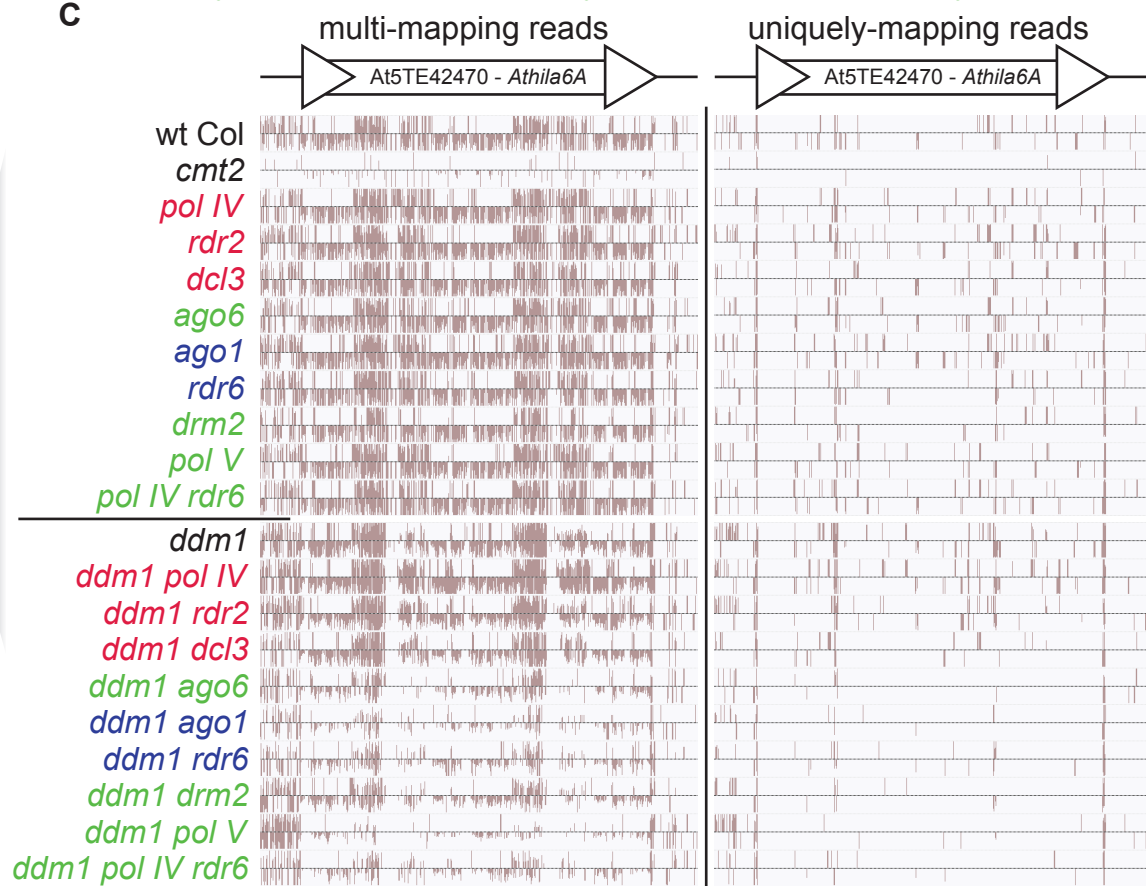

Supplement: Additional file 3: Figure S2. — MethylC-seq has the resolution to assay RDR6-RdDM on individual TE loci. Using our unique-mapping approach and our MethylC-seq dataset, the highly repetitive TE targets of RDR6-RdDM are assayable, which allows for locus-specific analysis of TE methylation patterns. (PDF 188 kb) [file 13059_2016_1032_MOESM3_ESM.pdf]

**Figure S3**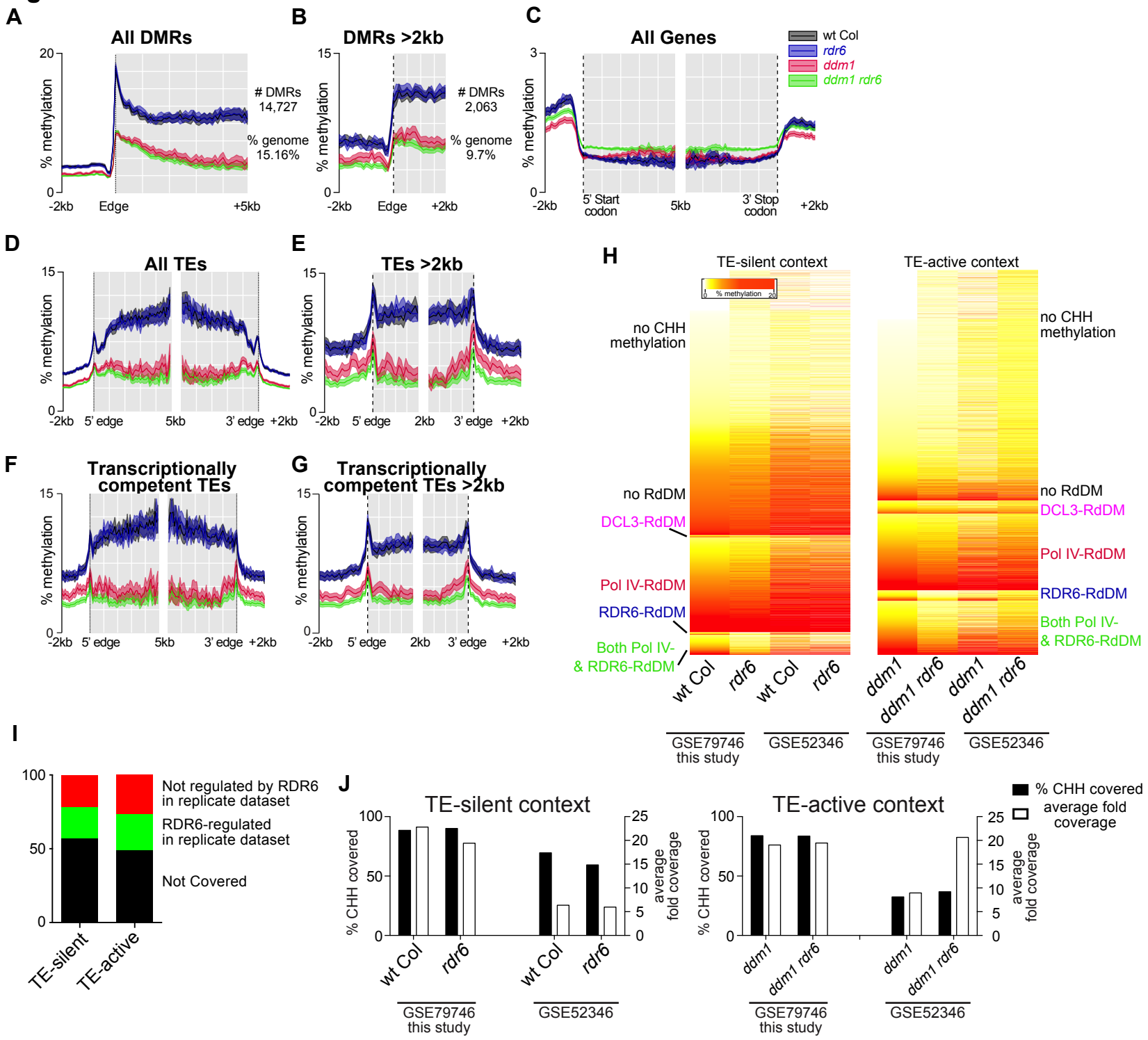

Supplement: Additional file 4: Figure S3. — Validation of MethylC-seq data using biological replicates of key genotypes. Analyses of biological replicates of key samples showing the reproducibility of major conclusions from our study. (PDF 317 kb) [file 13059_2016_1032_MOESM4_ESM.pdf]

**Figure S4****A**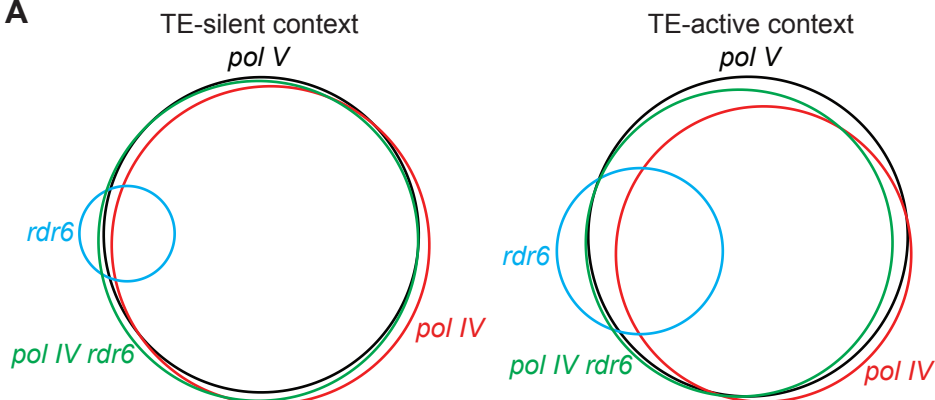**B**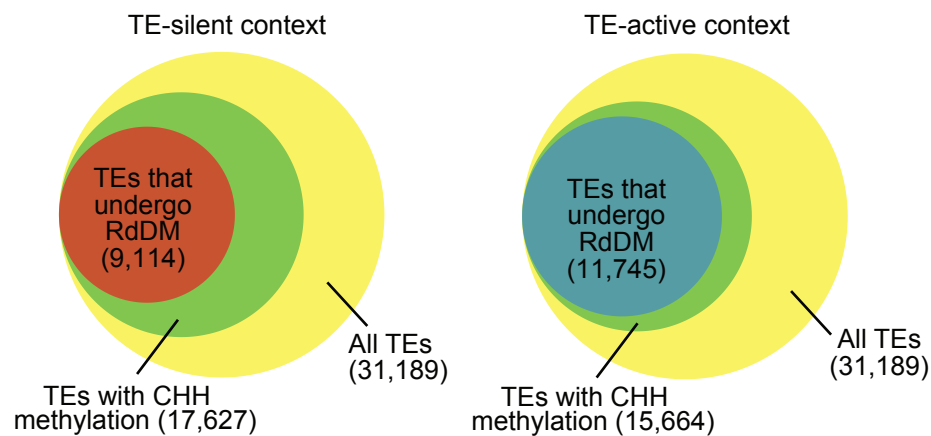**C**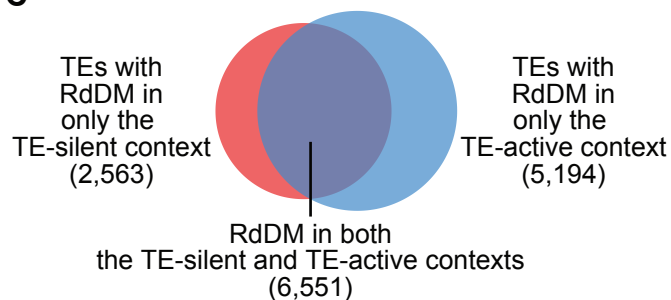**D**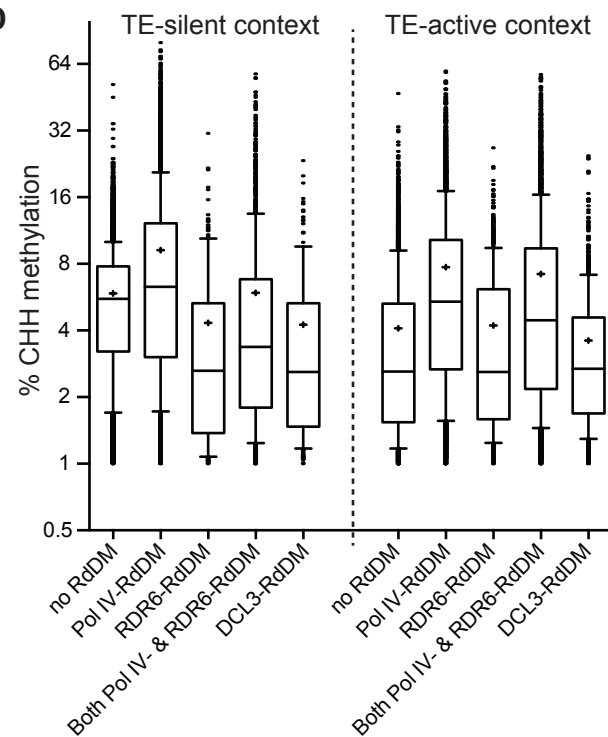

Supplement: Additional file 5: Figure S4. — Overlap in DMRs and TEs regulated by RdDM. Comparison of the relative efficacy of RdDM mechanisms in both TE-silent and TE-active contexts. (PDF 176 kb) [file 13059_2016_1032_MOESM5_ESM.pdf]

**Figure S5**

CG methylation for all assayable TEs (24,956/31,189)

**A**

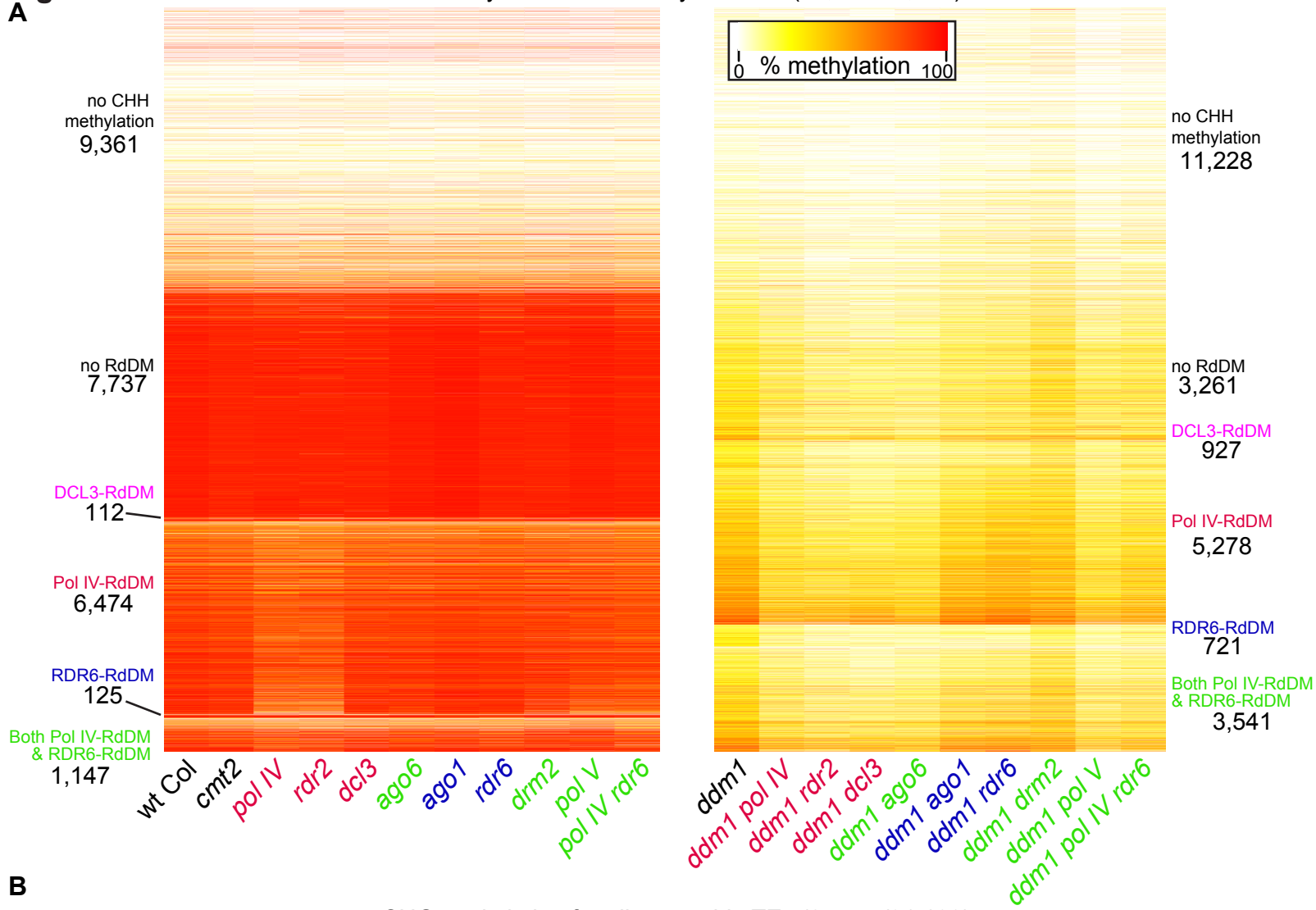

**B**

CHG methylation for all assayable TEs (25,457/31,189)

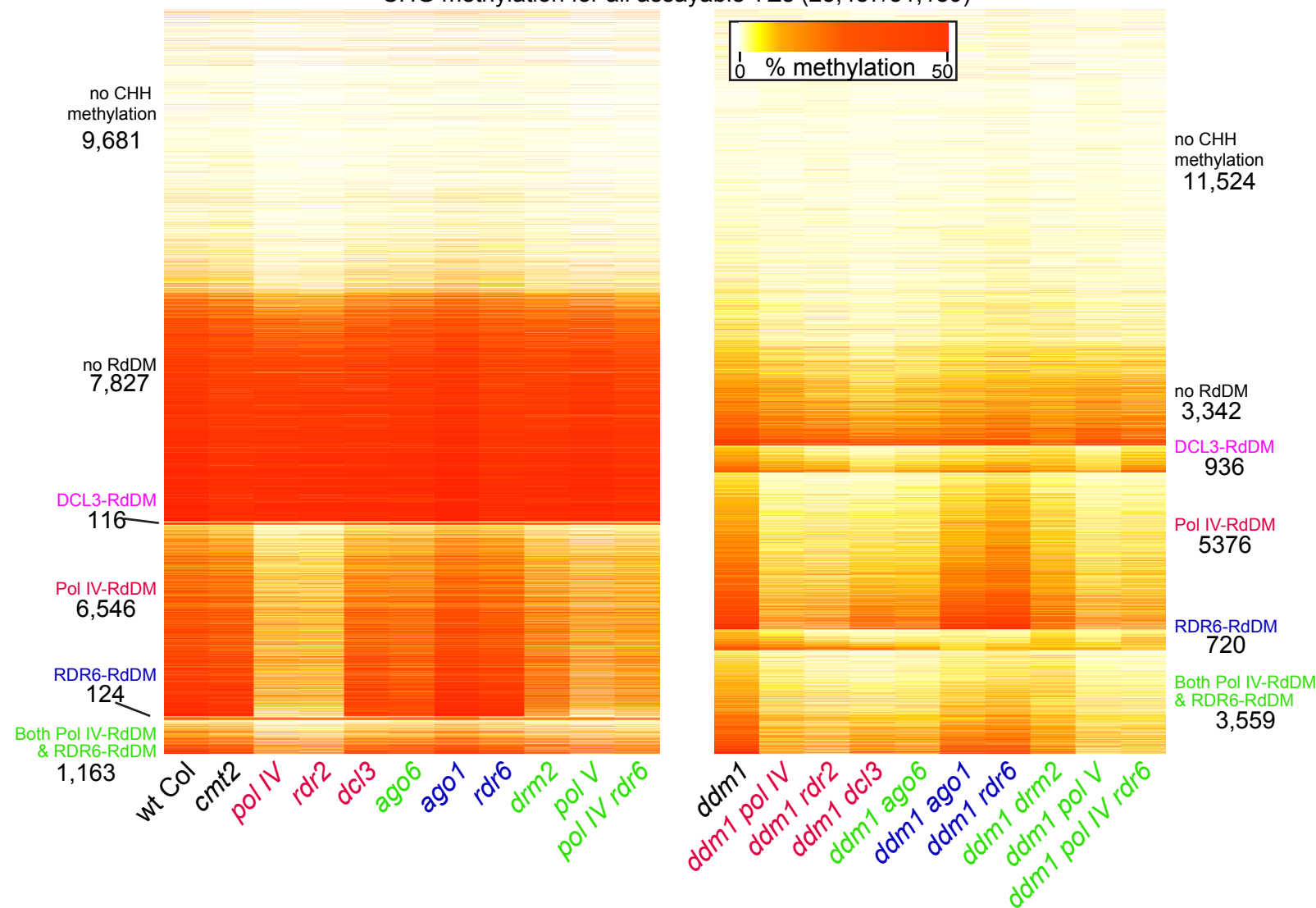

Supplement: Additional file 6: Figure S5. — Genome-wide distribution of TE CG and CHG methylation. Heatmap showing CG and CHG methylation for the TEs categorized into different RdDM mechanisms based on CHH methylation. (PDF 394 kb) [file 13059_2016_1032_MOESM6_ESM.pdf]

**Figure S7****A**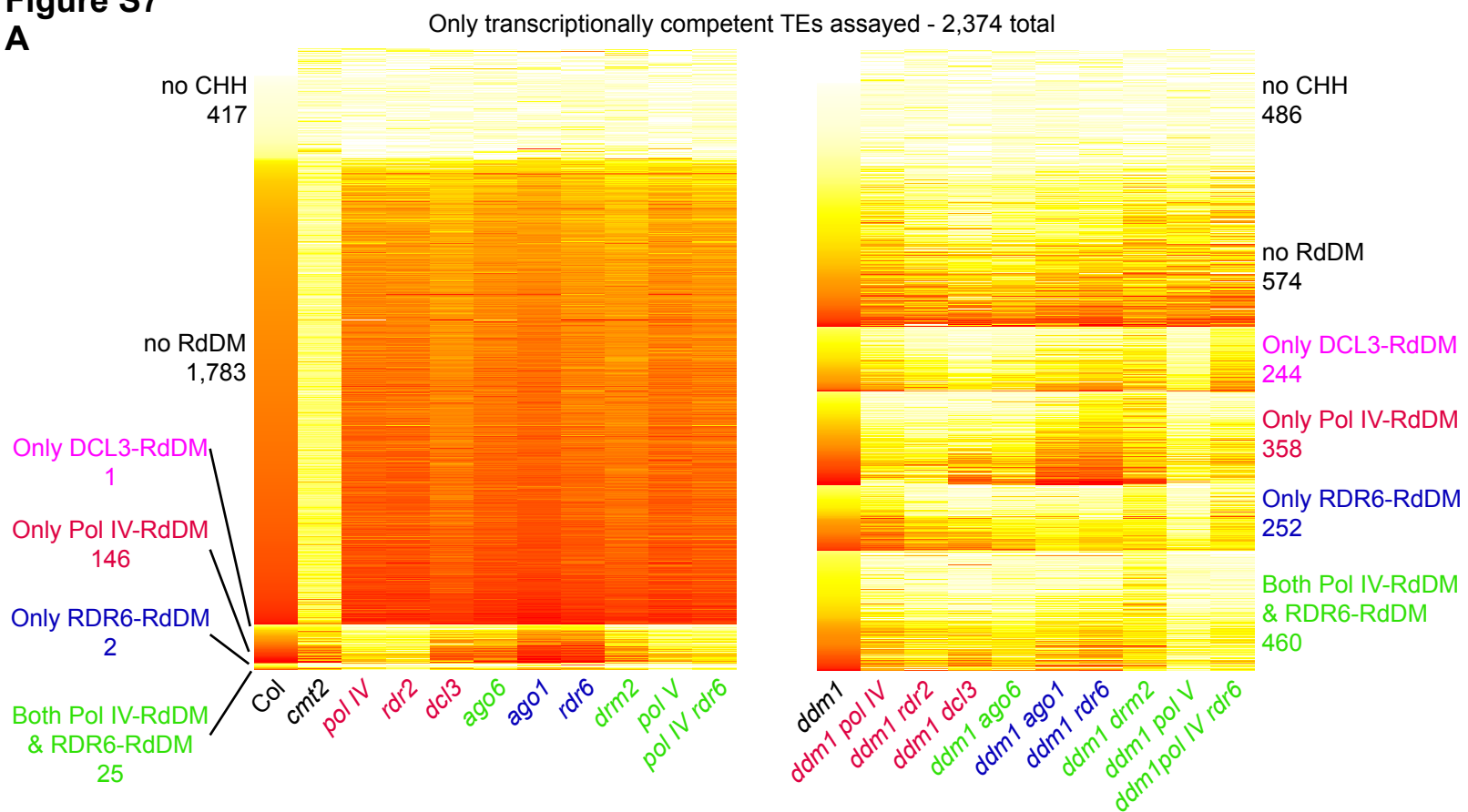**B**

Only transcriptionally competent LTR TEs assayed - 668 total

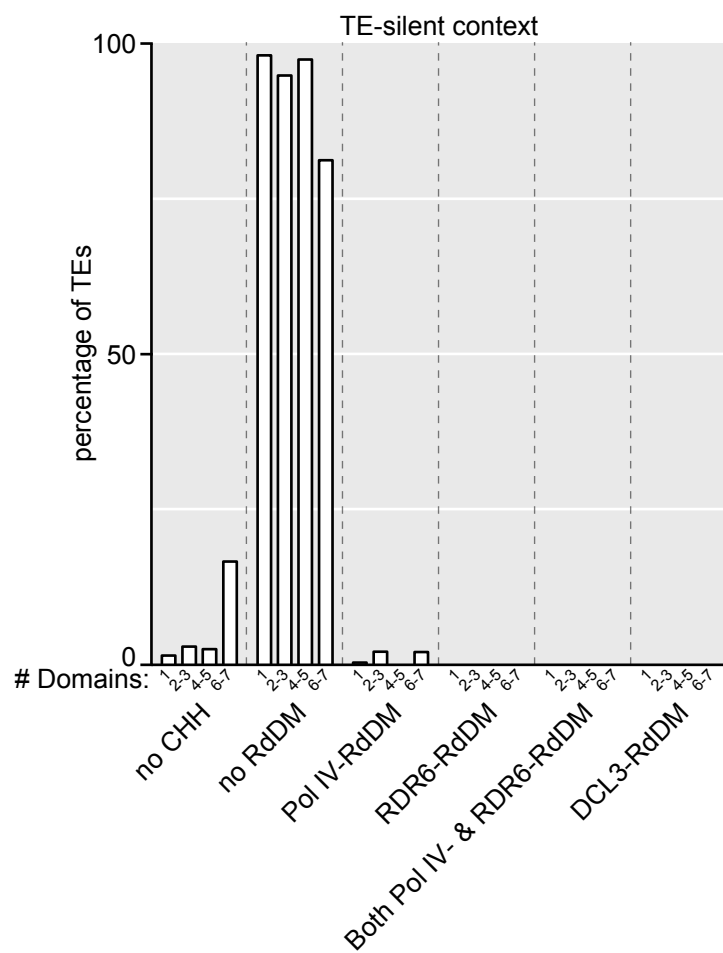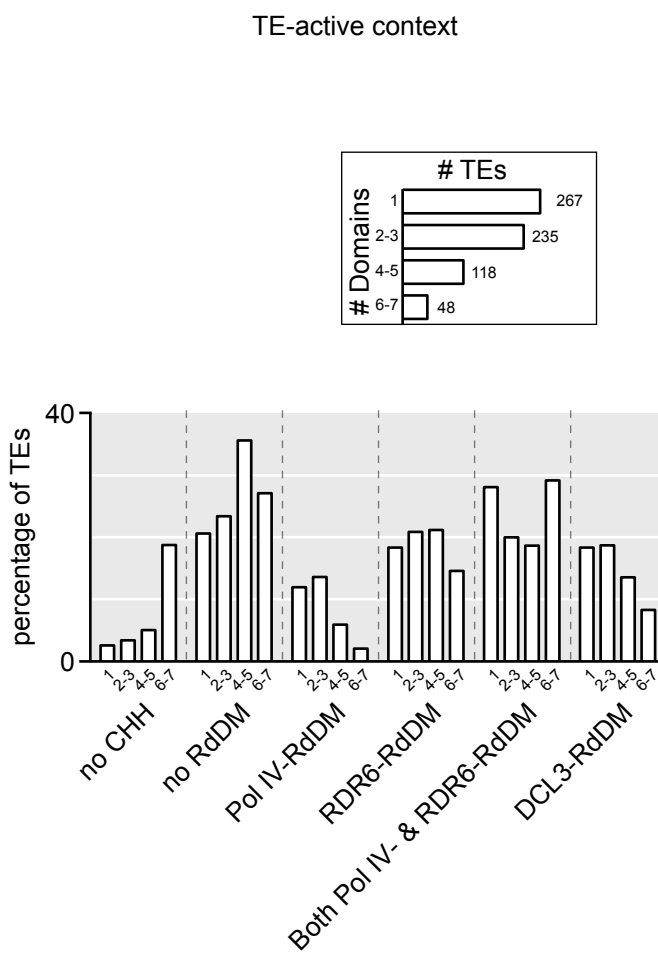

Supplement: Additional file 8: Figure S7. — Enrichment of RDR6-RdDM and DCL3-RdDM at transcriptionally competent TEs. We find that expression-dependent forms of RdDM are enriched when investigating the transcriptionally competent subset of TEs. (PDF 403 kb) [file 13059_2016_1032_MOESM8_ESM.pdf]

**Figure S8**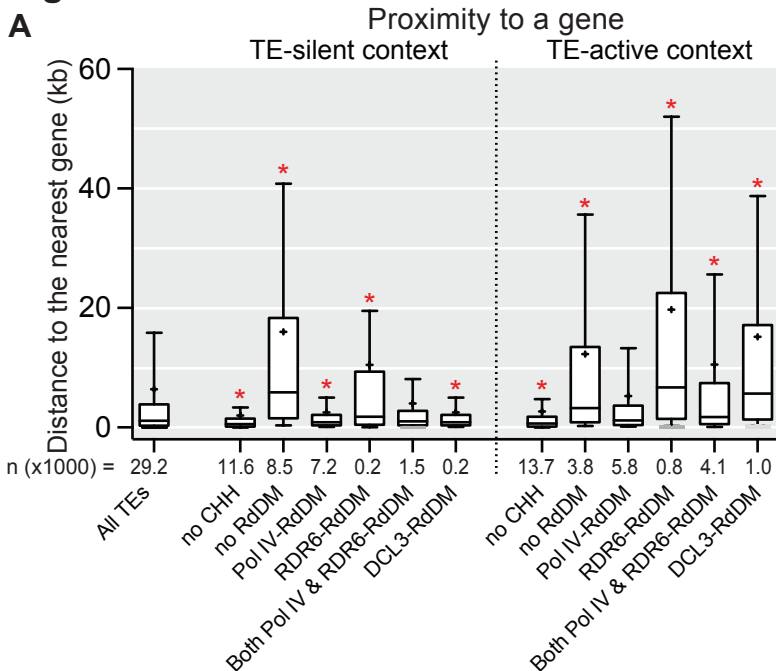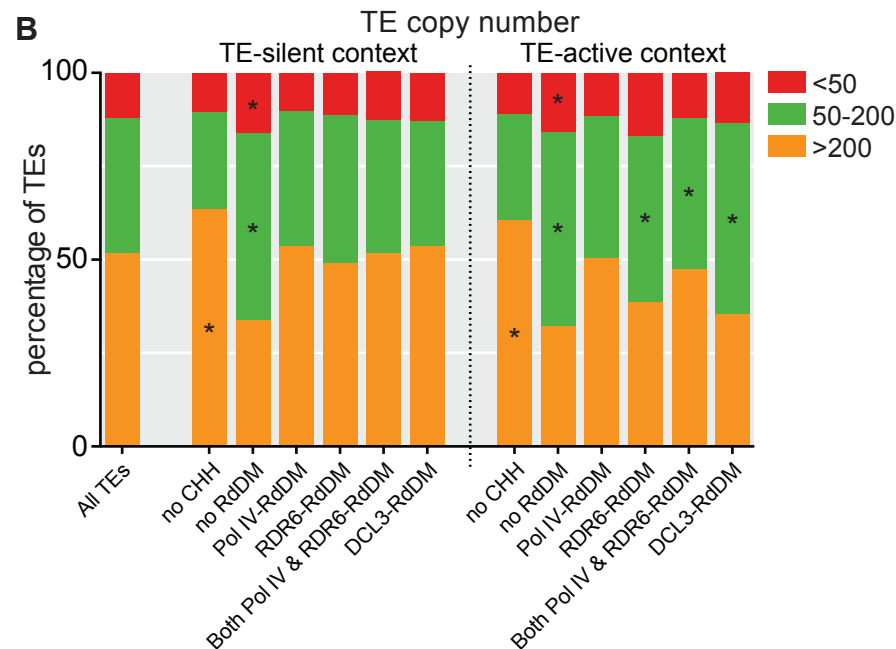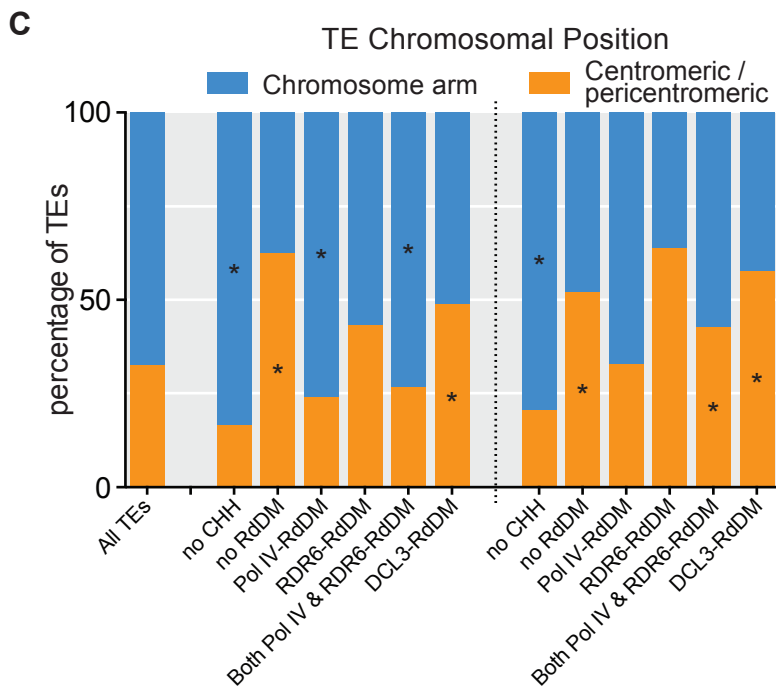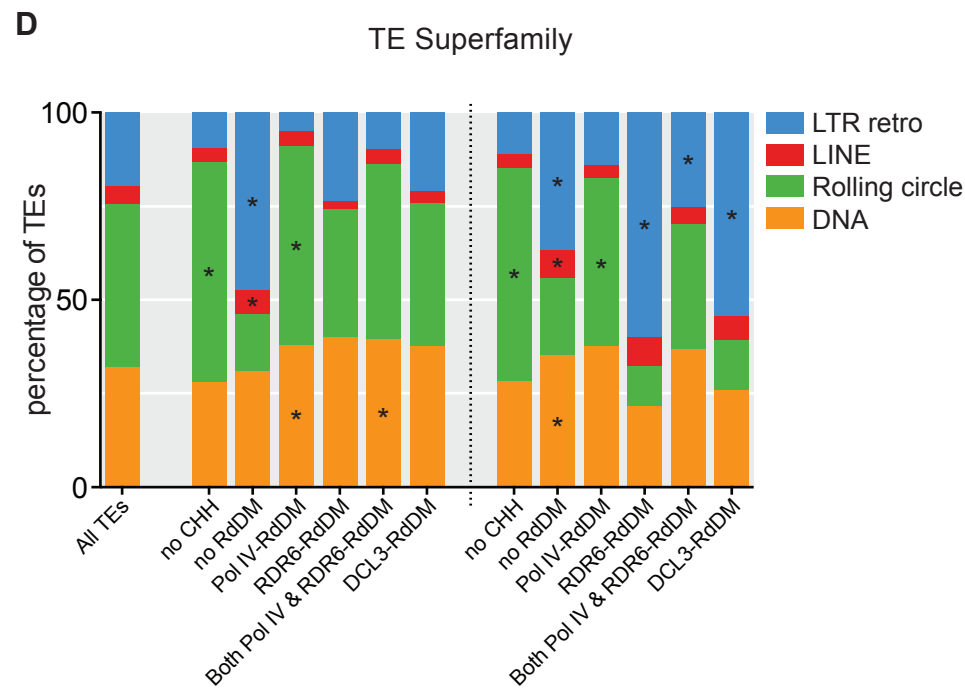

Supplement: Additional file 9: Figure S8. — Correlation between CHH methylation pathway and TE location, type, and copy number. Genome-wide trends exist for correlation of TE location, type, and copy number with the type of RdDM mechanism. (PDF 141 kb) [file 13059_2016_1032_MOESM9_ESM.pdf]
