# Supplementary material for: Full-length autonomous transposable elements are preferentially targeted by expression-dependent forms of RNA-directed DNA methylation
Source: Genome Biol. 2016 Aug 9;17:170. doi: 10.1186/s13059-016-1032-y (PMC4977677; doi:10.1186/s13059-016-1032-y)
Supplement: Additional file 2: — Results and Methods. Detailed Results, Methods, Figure Legends, and References to support the Supplemental Figures and Tables. (PDF 141 kb) [file 13059_2016_1032_MOESM2_ESM.pdf]

Additional file 2: Methods and Results

Supplemental Results

Arabidopsis TEs are individually assayable using uniquely mapping MethylC-seq reads

Previous publications have either used a uniquely-mapping strategy and avoided analysis of repetitive regions such as TEs, or taken a multi-mapping strategy and been unable to assay individual TEs [1,2]. The average CG, CHG and CHH methylation levels were determined for the whole genome, genes and TEs using both the multi-mapping and uniquely-mapping strategies (Additional file 3: Figure S2A). We found that although the coverage of TE loci is reduced using the uniquely-mapping strategy compared to multi-mapping (from 99.3% to 90.5% cytosines covered), the pattern of average methylation percentage was identical.

To determine if RDR6-RdDM could be visualized in the uniquely-mapping dataset, we investigated long (over 2,500bp) *Athila* LTR retrotransposon TEs, a known target of RDR6-RdDM in the *ddm1* background [3]. We identified a reduction from 8.8% CHH methylation in the *ddm1* single mutant to 3.2% in the *ddm1 rdr6* double mutant for both the multi-mapping and uniquely-mapping approaches (inset graph, Additional file 3: Figure S2A), demonstrating that TE RDR6-RdDM can be assayed using uniquely-mapping MethylC-seq reads for even high copy TEs.

Further interrogation of an individual *Athila6A* TE copy (At5TE42470) with a low mappability score (0.52)(Additional file 3: Figure S2B) demonstrates that even with the low mappability of MethylC-seq reads and reduced coverage associated with uniquely mapping reads, RDR6-RdDM at a high-copy TE is detected and confirmed: in the *ddm1* TE-active context CHH methylation is dependent on RDR6, AGO1, Pol V, AGO6 and DRM2 (Additional file 3: Figure S2C) [4].

Dicer-dependent and independent RdDM

To demonstrate that nearly all Pol II-mediated RdDM is lost in the *rdr6* mutant and that a *pol IV rdr6* double mutant removes the vast majority of RdDM, we compared the overlap in DMRs between *pol V*, *pol IV*, *rdr6* and *pol IV rdr6* (Additional file 5: Figure S4A). Pol V DMRs represent the set of all known RdDM targets (8,194 in the TE-silent context, 10,421 in TE-active context). The percent of Pol V DMR overlap in the TE-silent context raises from 95.4% in *pol IV* to 95.8% in *pol4 rdr6*, and in the TE-active context from 80% in *ddm1 pol IV* to 85.5% in *ddm1 pol IV rdr6* (Additional file 5: Figure S4A).

<u>Supplemental Methods</u>

Mappability

We used the program *GEM-mappability* [5] to calculate the mappability of TEs. For small RNAs we used a k-mer size of 24nt with 0 mismatches. As a control for our bisulfite sequencing read mappability, we used a k-mer size of 150nt with 2 mismatches. For calculating the bisulfite sequencing read mappability, we altered the sequence of the Arabidopsis genome (depending on whether or not a specific cytosine is methylated). After this alteration (*in silico*-bisulfite conversion) of the genome, we calculated the mappability of all TEs. The mappability of an individual TE is shown as the average mappability of all its nucleotides in Additional file 3: Figure S2B.

TE categorization based on size, copy number and location

We calculated the length of TEs based on the start and stop position in the TAIR10 annotation.

The TEs were divided into four categories based on their length: long (>5kb), medium (2-5kb), short (0.1-2kb) and tiny (<0.1 kb). To determine the distance of the TEs from the nearest gene, we calculated the closest annotated non-TE gene. To determine the copy number of a TE, we calculated the number of copies of the specific TE sub-family and categorized each TE into one of three categories: high copy (>200); medium copy (50-200) and low copy (<50). To determine the position of a TE on a chromosome, the pericentromeric region was defined as in [6].

RNA-seq analysis

For expression analysis, we used two publically available datasets: GSE38286 and GSE67799, which both investigated seedling tissues. We used *bowtie* to map the RNA-seq reads using the parameters *-v 2 -m 1* (only unique mapping sequences, up to two mismatches allowed) and calculated RPKM for each TE (see section on sRNA mapping). For Additional file 1: Figure S1, we used the function *beanplot* (kernel = *gaussian*) from package *beanplot* in *R* to plot the RPKM density.

Supplemental Figure Legends

Additional file 1: Figure S1. Steady-state TE mRNA accumulation in *ddm1* and the RdDM mutant *pol V*

TE steady-state mRNA accumulation in wt Col, *ddm1* and the *pol V* (*nrpe1*) mutant seedlings from GSE38286 and GSE67799. Mutants are compared to their respective wt Col control datasets. *pol V* mutants were chosen for this analysis because the level of RdDM and CHH methylation are lowest in this genotype (Figure 2E). Full-length TEs are more efficiently silenced in wt Col compared to TE fragments, and undergo a larger shift in reactivation in *ddm1* mutants. The activation of TEs in *pol V* mutants is minor compared to the reactivation that occurs in *ddm1*.

Additional file 3: Figure S2. MethylC-seq has the resolution to assay RDR6-RdDM on individual TE loci.

A. Bar graphs comparing the percent methylation using multi-mapping (top) and uniquely-mapping (bottom) approaches. The percent of cytosines not covered is shown in parentheses. The inset displays the change in CHH methylation between *ddm1* and *ddm1 rdr6* for long (>2,500bp) *Athila6A* TEs.

B. Average unique mappability of each TE in the genome for unconverted wt DNA (wt Col), *in silico* bisulfite converted wt Col DNA (wt Col BS) and *ddm1* TE-active *in silico* bisulfite converted DNA (*ddm1* BS). The *Athila6A* TE At5TE42470 has a low mappability score and is shown in detail in part C.

C. CHH methylation levels of the RDR6-RdDM targeted TE *Athila6A* at the specific locus At5TE42470 using both multi-mapping and uniquely-mapping approaches. In the TE-silent context, the methylation of this TE is maintained via CMT2. Upon transcriptional activation, the methylation detected in *ddm1* single mutants is contributed by primarily RDR6-RdDM.

Genotypes are color-coded based on the methylation pathway (black = maintenance methylation, red = Pol IV-RdDM, blue = RDR6-RdDM, green = contributes to both Pol IV-RdDM and RDR6-RdDM). In part C the scale of CHH methylation is from 0 to 33.3%, while bars above and below the dotted line represent the top and bottom strands of DNA.

Additional file 4: Figure S3. Validation of MethylC-seq data using biological replicates of key genotypes

MethylC-seq data for the same tissue of wt Col, *rdr6*, *ddm1* and *ddm1 rdr6* are publically available from GSE52346 [2]. Although the sequencing approach is distinct, and this introduces technical variation, reanalysis of this raw data demonstrates that our major conclusions in Figures 1 and 2 are reproducible.

A. CHH methylation levels from GSE52346 at the DMRs identified in Figure 1 (using the data produced in this study).

B. DMRs over 2.0kb from Figure 1 with data from GSE52346.

C. CHH methylation level from GSE52346 over all genes.

D. CHH methylation level from GSE52346 over all TEs.

E. CHH methylation level from GSE52346 across TEs >2.0kb.

F. CHH methylation level from GSE52346 over the set of transcriptionally competent TEs identified in Figure 1.

G. CHH methylation level from GSE52346 over the set of transcriptionally competent TEs >2.0kb.

H. Heat map comparing TE CHH methylation levels from this study to those identified in GSE52346. The distribution of TEs into RdDM pathways was performed based on the data in this study and is the same as in Figure 2.

I. The number of TEs identified as targeted by RDR6-RdDM from the dataset produced in this study, compared to the GSE52346 dataset. Most of RDR6-RdDM TEs are not covered by uniquely mapping sequencing reads in the GSE52346 dataset. Of the TEs that are covered in both datasets, ~50% of TEs have RDR6-dependent CHH methylation in both datasets.

J. Coverage of cytosines from covered but not replicated TEs from part I in our dataset and GSE52346. Both the percent of CHH sites covered (left Y-axis) and average fold coverage (right Y-axis) are calculated for wt Col, *rdr6, ddm1* and *ddm1 rdr6* from both datasets.

For analysis of GSE52346 data, we re-mapped raw reads using the uniquely mapping approach outlined in the Methods section.

Additional file 5: Figure S4. Overlap in DMRs and TEs regulated by RdDM

A. Venn diagram of number of DMRs in *pol V*, *pol IV*, *rdr6* and *pol IV rdr6* mutants in both the TE-silent and TE-active contexts. See the Supplemental Results section for DMR numbers.

B. Venn diagram of the number of TEs with CHH methylation that undergo any type of RdDM in TE-silent compared to TE-active contexts.

C. Venn diagram of the overlap of TEs that undergo any type of RdDM in TE-silent compared to TE-active contexts.

D. CHH methylation level of the TE targets of each indicated pathway. Box plots represent the median and interquartile range, and whiskers are the 10-90% range. The plus sign indicates the mean value. Pol IV-RdDM target TEs have a statistically higher ($p<0.001$, one-way Anova) level of CHH methylation compared to RDR6-RdDM and DCL3-RdDM.

Additional file 6: Figure S5. Genome-wide distribution of TE CG and CHG methylation

A. Heatmap of CG DNA methylation of TEs.

B. Heatmap of CHG DNA methylation of TEs.

Each TE represents a horizontal row, while the columns represent methylation levels in different mutant genotypes. The categorization into different RdDM pathways is based on the CHH methylation from Figure 2E. TEs change position between the TE-silent (left) and *ddm1* TE-active (right) panel. Color-coding of genotypes is based on Figure 2E.

Additional file 7: Figure S6. Pol IV- and RDR6-RdDM compensate for each other

A. Number of TEs with CHH methylation dependent on either Pol IV (green) or RDR6 (purple) in the TE-silent and TE-active contexts. This data was determined using only the *pol IV* and *rdr6* single mutants in the TE-silent or TE active context.

B. The number of TEs regulated by both Pol IV- and RDR6-RdDM when both Pol IV- and RDR6-RdDM are mutated at the same time (in the *pol IV rdr6* double mutant). The number of TEs co-regulated by both Pol IV- and RDR6-RdDM (overlap in Venn diagram) increases when the *pol IV rdr6* double mutant is analyzed compared to when only the single mutants are analyzed (see part A). This demonstrates that there are some TEs that both Pol IV- and RDR6-RdDM target, and loss of CHH methylation can only be detected from these TEs when both pathways are lost at the same time (particularly in the TE-active context).

Additional file 8: Figure S7. Enrichment of RDR6-RdDM and DCL3-RdDM at transcriptionally competent TEs

A. Heatmap of CHH DNA methylation of the set of 2,374 transcriptionally competent TEs. TEs change position between the TE-silent (left) and *ddm1* TE-active (right) panels. Most transcriptionally competent TEs are not targeted by RdDM, but rather targeted by CMT2-based maintenance CHH methylation in the TE-silent context, and then distribute to a RdDM pathway

when transcriptionally activated. The Pol II expression-dependent pathways of RDR6-RdDM and DCL3-RdDM are particularly enriched compared to the analysis of all TEs in Figure 2E. Genotypes are color-coded based on Figure 2E.

B. The RdDM categorization of transcriptionally competent LTR retrotransposons based on the number of domains essential for LTR retrotransposition listed in Figure 5A. Inset graph displays the total number of LTR retrotransposons in each category.

Additional file 9: Figure S8. Correlation between CHH methylation pathway and TE location, type and copy number

A. Distance to the nearest gene distribution of TEs via their RdDM pathway in the TE-silent or TE-active context. Box plot whiskers represent 10th-90th percentile while the mean is shown as a plus sign. Asterisks represent statistical significance difference of p<0.001 in a one-way Anova test compared to the total TE distribution.

B. Copy number distribution of TEs via their CHH methylation pathway in the TE-silent or TE-active context.

C. TE chromosomal position distribution via their CHH methylation pathway in the TE-silent or TE-active context.

D. TE superfamily distribution via their CHH methylation pathway in the TE-silent or TE-active context.

Asterisks in parts B-D represent statistical significance of p<0.001 using a Chi-squared test of homogeneity followed by a multiple comparison test for unequal sample sizes. The number of TEs displayed for the different RdDM categories in part A are the same for the other parts of this figure.

Additional file 10: Figure S9. TE cleavage dynamics

A. Percentage of LTR retrotransposons with cleaved mRNAs in the TE-silent and TE-active contexts.

B. Percentage of type I non-LTR and type II TEs with cleaved mRNAs in the TE-silent and TE-active contexts.

C. Analysis of the mRNA accumulation level and siRNA production from the TEs that have the same cleavage sites in both the TE-active and TE-silent contexts. Steady-state mRNA production is mined from the same dataset in Additional file 1: Figure S1. siRNA production refers to prefect and uniquely matching siRNAs. TEs with the same cleavage site in the TE-silent and TE-active contexts increase in mRNA accumulation in the *ddm1* mutant background, however do not produce abundant secondary siRNAs.

D. Size distribution of the siRNAs responsible for cleaving the “Cleavage sites not retained & completely distinct in *ddm1*” TEs from Figure 6G. A shift in the size distribution was not detected for the small RNAs responsible for triggering secondary siRNA production only in the *ddm1* TE-active context.

Supplemental References

1. Lister R, Pelizzola M, Dowen RH, Hawkins RD, Hon G, Tonti-Filippini J, et al. Human DNA methylomes at base resolution show widespread epigenomic differences. Nature. 2009;462:315–22.

2. Creasey KM, Zhai J, Borges F, Van Ex F, Regulski M, Meyers BC, et al. miRNAs trigger widespread epigenetically activated siRNAs from transposons in Arabidopsis. Nature. 2014;508:411–5.

3. Nuthikattu S, McCue AD, Panda K, Fultz D, Defraia C, Thomas EN, et al. The Initiation of Epigenetic Silencing of Active Transposable Elements is Triggered by RDR6 and 21-22 Nucleotide Small Interfering RNAs. Plant Physiol. 2013;162:116–31.

4. McCue AD, Panda K, Nuthikattu S, Choudury SG, Thomas EN, Slotkin RK. ARGONAUTE 6 bridges transposable element mRNA-derived siRNAs to the establishment of DNA methylation. EMBO J. 2014;34:20–35.

5. Derrien T, Estellé J, Sola SM, Knowles DG, Raineri E, Guigó R, et al. Fast Computation and Applications of Genome Mappability. PLoS ONE. 2012;7:e30377.

6. Kawabe A, Hansson B, Hagenblad J, Forrest A, Charlesworth D. Centromere locations and associated chromosome rearrangements in Arabidopsis lyrata and A. thaliana. Genetics. 2006;173:1613–9.
