# Supplementary material for: Full-length autonomous transposable elements are preferentially targeted by expression-dependent forms of RNA-directed DNA methylation
Source: Genome Biol. 2016 Aug 9;17:170. doi: 10.1186/s13059-016-1032-y (PMC4977677; doi:10.1186/s13059-016-1032-y)
Supplement: Additional file 7: Figure S6. — Pol IV- and RDR6-RdDM compensate for each other. Evidence of RdDM compensation is observed when both Pol IV and RDR6 are mutated simultaneously in either TE-silent or TE-active context. (PDF 105 kb) [file 13059_2016_1032_MOESM7_ESM.pdf]

Figure S6

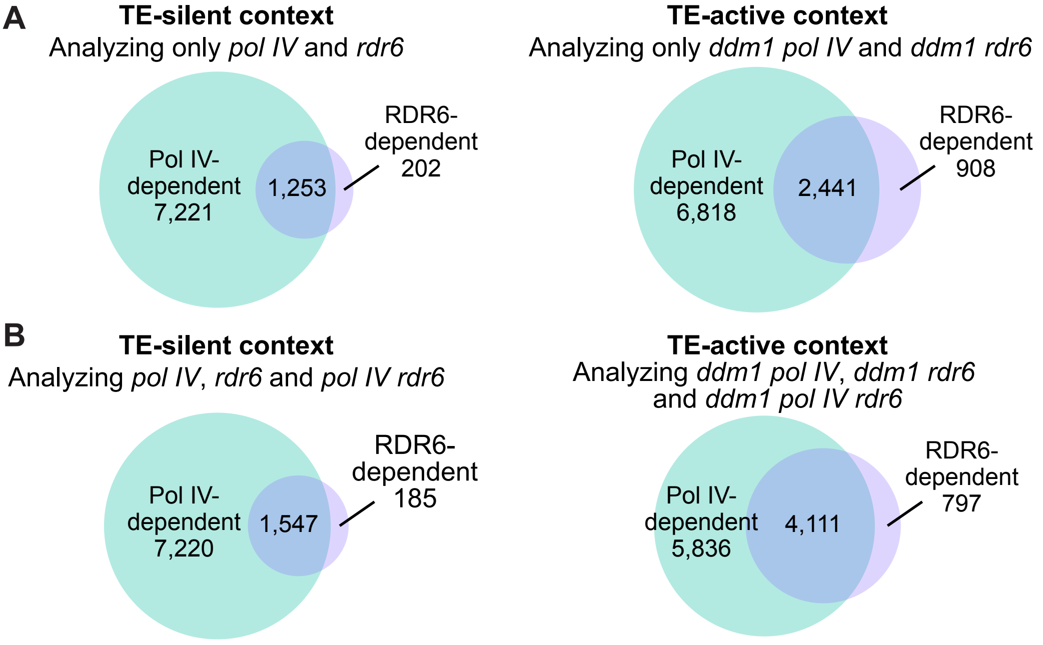
A
TE-silent context
Analyzing only pol IV and rdr6
Pol IV-dependent 7,221
1,253
RDR6-dependent 202
TE-active context
Analyzing only ddm1 pol IV and ddm1 rdr6
Pol IV-dependent 6,818
2,441
RDR6-dependent 908
B
TE-silent context
Analyzing pol IV, rdr6 and pol IV rdr6
Pol IV-dependent 7,220
1,547
RDR6-dependent 185
TE-active context
Analyzing ddm1 pol IV, ddm1 rdr6 and ddm1 pol IV rdr6
Pol IV-dependent 5,836
4,111
RDR6-dependent 797
