# Supplementary material for: Full-length autonomous transposable elements are preferentially targeted by expression-dependent forms of RNA-directed DNA methylation
Source: Genome Biol. 2016 Aug 9;17:170. doi: 10.1186/s13059-016-1032-y (PMC4977677; doi:10.1186/s13059-016-1032-y)
Supplement: Additional file 10: Figure S9. — TE cleavage dynamics. Cleavage site analyses demonstrate that size of the primary small RNAs and TE mRNA accumulation level do not dictate secondary siRNA production. (PDF 153 kb) [file 13059_2016_1032_MOESM10_ESM.pdf]

Figure S9

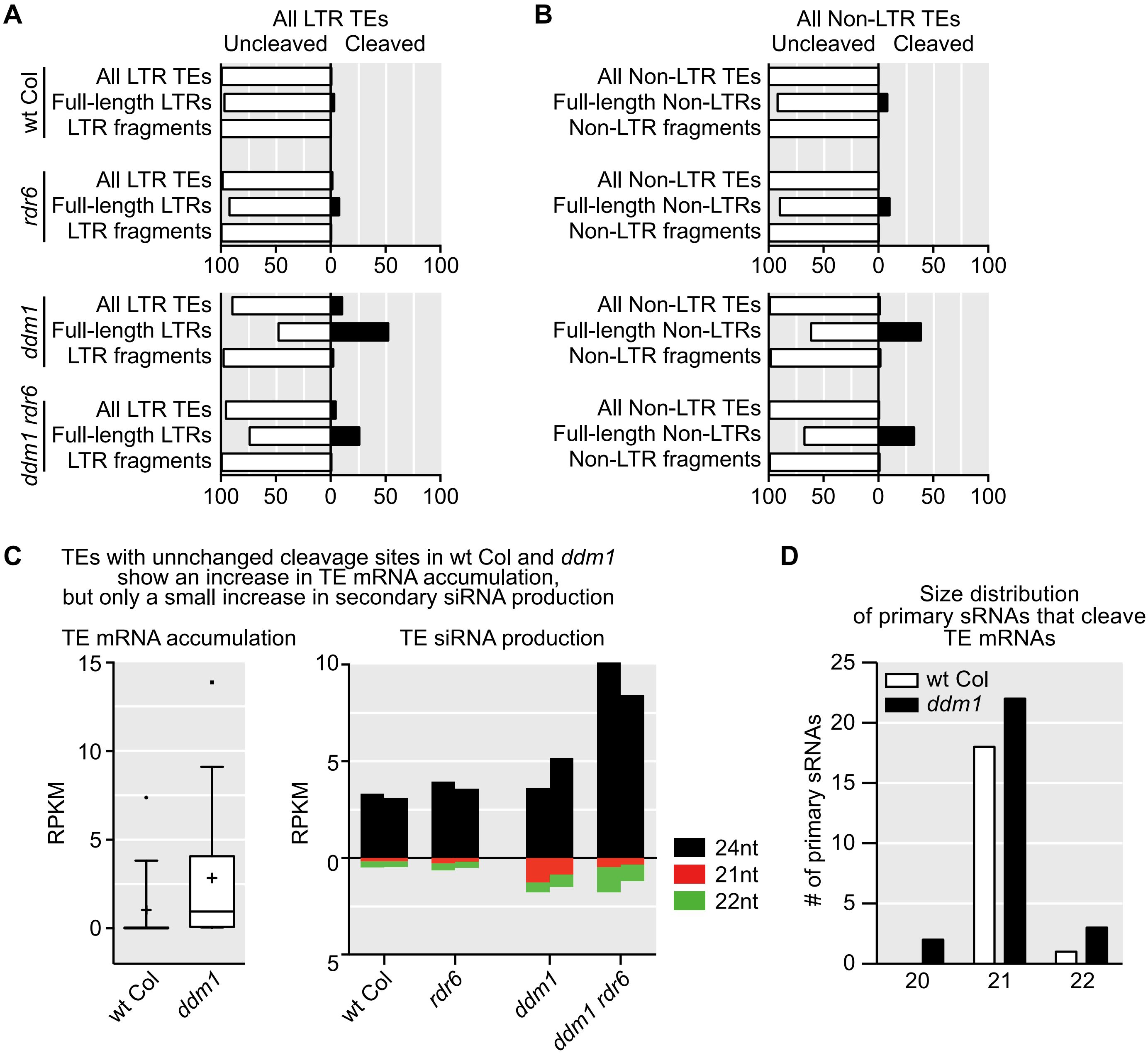
A
All LTR TEs
Uncleaved Cleaved
wt Col
rdr6
ddm1
ddm1 rdr6
All LTR TEs
Full-length LTRs
LTR fragments
100 50 0 50 100
B
All Non-LTR TEs
Uncleaved Cleaved
All Non-LTR TEs
Full-length Non-LTRs
Non-LTR fragments
100 50 0 50 100
C
TEs with unnchanged cleavage sites in wt Col and ddm1 show an increase in TE mRNA accumulation, but only a small increase in secondary siRNA production
TE mRNA accumulation
RPKM
0 5 10 15
wt Col
ddm1
TE siRNA production
RPKM
5 0 5 10
wt Col
rdr6
ddm1
ddm1 rdr6
24nt
21nt
22nt
D
Size distribution of primary sRNAs that cleave TE mRNAs
# of primary sRNAs
0 5 10 15 20 25
wt Col
ddm1
20 21 22
