# Supplementary material for: Full-length autonomous transposable elements are preferentially targeted by expression-dependent forms of RNA-directed DNA methylation
Source: Genome Biol. 2016 Aug 9;17:170. doi: 10.1186/s13059-016-1032-y (PMC4977677; doi:10.1186/s13059-016-1032-y)
Supplement: Additional file 11: Table S1. — Mutant alleles and quality statistics of the MethylC-seq data produced in this study. Reads from our MethylC-seq dataset cover more than 96 % of all cytosines in the genome with a roughly 25-fold coverage. (PDF 48 kb) [file 13059_2016_1032_MOESM11_ESM.pdf]

Table S1

ID	Name	Allele	Read size	% Non-conversion	Mapped reads	%	Genome coverage	Mapped cytosines	%	Genome-wide methylation level (uniquely mapped data) mCG	mCHG	mCHH
1	wt Col	-	150	0.28%	19,989,099	55.38%	25.13	41,481,405	96.78%	22.0%	7.4%	1.3%
2	*cmt2*	*Salk_012874*	150	0.38%	16,928,589	57.16%	21.28	41,266,163	96.28%	21.1%	6.7%	0.7%
3	*pol IV*	*Salk_128428*	150	0.27%	15,676,003	56.91%	19.71	41,325,833	96.42%	24.1%	7.2%	1.1%
4	*rdr2*	*rdr2-1*	150	0.27%	20,397,486	51.80%	25.65	41,418,932	96.64%	23.8%	7.1%	1.1%
5	*dcl3*	*dcl3-1*	150	0.28%	18,239,145	57.44%	22.93	41,447,723	96.71%	21.6%	6.7%	1.1%
6	*ago6*	*ago6-2*	150	0.31%	14,687,638	52.82%	18.47	41,202,531	96.13%	24.8%	8.3%	1.4%
7	*ago1*	*ago1-27*	150	0.26%	22,797,974	58.67%	28.66	41,530,662	96.90%	23.8%	8.6%	1.6%
8	*rdr6*	*rdr6-15*	150	0.31%	17,393,333	56.04%	21.87	41,375,083	96.54%	22.3%	7.2%	1.4%
9	*drm2*	*Salk_150863*	150	0.34%	17,154,747	59.27%	21.57	41,239,830	96.22%	21.1%	6.2%	1.0%
10	*pol V*	*Salk_017795*	150	0.26%	12,026,143	37.31%	15.12	41,133,677	95.97%	25.1%	8.1%	1.2%
11	*pol IV rdr6*	-	150	0.27%	18,898,806	54.50%	23.76	41,450,148	96.71%	22.1%	6.3%	1.0%
12	*ddm1*	*ddm1-2*	150	0.22%	21,290,878	51.11%	26.77	41,506,256	96.84%	12.4%	3.6%	1.1%
13	*ddm1 pol IV*	-	150	0.28%	23,548,653	56.84%	29.61	41,408,027	96.61%	11.8%	2.7%	0.8%
14	*ddm1 rdr2*	-	150	0.28%	25,729,839	55.14%	32.35	41,630,586	97.13%	9.8%	2.7%	0.7%
15	*ddm1 dcl3*	-	150	0.22%	16,161,892	55.57%	20.32	41,415,534	96.63%	9.5%	2.1%	0.6%
16	*ddm1 ago6*	-	150	0.28%	17,335,248	53.65%	21.80	41,524,746	96.89%	9.5%	2.0%	0.6%
17	*ddm1 ago1*	-	150	0.27%	19,484,305	57.29%	24.50	41,465,759	96.75%	10.6%	2.2%	0.9%
18	*ddm1 rdr6*	-	150	0.26%	25,164,121	56.93%	31.64	41,510,183	96.85%	11.1%	2.8%	1.0%
19	*ddm1 drm2*	-	150	0.27%	20,053,406	58.21%	25.21	41,445,830	96.70%	13.2%	2.3%	0.8%
20	*ddm1 pol V*	-	150	0.29%	27,917,845	53.37%	35.10	41,550,703	96.95%	10.7%	2.5%	0.6%
21	*ddm1 pol IV rdr6*	-	150	0.25%	22,188,729	54.74%	27.90	41,450,339	96.71%	11.1%	2.2%	0.7%
	Average			0.28%	19,669,708.52	54.77%	24.73	41,418,092.86	96.64%	17.2%	5.0%	1.0%
