# Supplementary material for: Full-length autonomous transposable elements are preferentially targeted by expression-dependent forms of RNA-directed DNA methylation
Source: Genome Biol. 2016 Aug 9;17:170. doi: 10.1186/s13059-016-1032-y (PMC4977677; doi:10.1186/s13059-016-1032-y)
Supplement: Additional file 12: Table S2. — Primer sequences used in this report. Primer sequences for single-locus bisulfite sequencing performed in Fig. 3c, d are reported. (PDF 36 kb) [file 13059_2016_1032_MOESM12_ESM.pdf]

Table S2
Primer sequences used in this report

Target	Experiment	Figure	Forward primer sequence	Reverse primer sequence
At5g52070 red box	single-locus bisulfite sequencing	Figure 3C	ATTGATGGAGTAGATGGGGTGGT	TCCAATTATCTTRCTTCAAAACCAA
At3TE40740	single-locus bisulfite sequencing	Figure 3D	GAGAATTATTAAAYAGAYGTATGGG	CTTTAACRTTAAAARCCCATTAT

Key for degenerate bases: M=A/C, R=A/G, W=A/T, S=C/G, Y=C/T, K=G/T
